# fNIRS a novel neuroimaging tool to investigate olfaction, olfactory imagery, and crossmodal interactions: a systematic review

**DOI:** 10.3389/fnins.2024.1266664

**Published:** 2024-01-31

**Authors:** Eleanor Boot, Andrew Levy, Giuliano Gaeta, Natalie Gunasekara, Emilia Parkkinen, Emily Kontaris, Muriel Jacquot, Ilias Tachtsidis

**Affiliations:** ^1^Metabolight Ltd., London, United Kingdom; ^2^Wellcome Centre for Human Neuroimaging, University College, London, United Kingdom; ^3^Health and Well-being Centre of Excellence, Givaudan UK Limited, Ashford, United Kingdom; ^4^Department of Medical Physics and Biomedical Engineering, University College London, London, United Kingdom

**Keywords:** olfaction, olfactory imagery, crossmodal visual-olfactory integration, systematic review, neuroimaging, fNIRS

## Abstract

Olfaction is understudied in neuroimaging research compared to other senses, but there is growing evidence of its therapeutic benefits on mood and well-being. Olfactory imagery can provide similar health benefits as olfactory interventions. Harnessing crossmodal visual-olfactory interactions can facilitate olfactory imagery. Understanding and employing these cross-modal interactions between visual and olfactory stimuli could aid in the research and applications of olfaction and olfactory imagery interventions for health and wellbeing. This review examines current knowledge, debates, and research on olfaction, olfactive imagery, and crossmodal visual-olfactory integration. A total of 56 papers, identified using the PRISMA method, were evaluated to identify key brain regions, research themes and methods used to determine the suitability of fNIRS as a tool for studying these topics. The review identified fNIRS-compatible protocols and brain regions within the fNIRS recording depth of approximately 1.5 cm associated with olfactory imagery and crossmodal visual-olfactory integration. Commonly cited regions include the orbitofrontal cortex, inferior frontal gyrus and dorsolateral prefrontal cortex. The findings of this review indicate that fNIRS would be a suitable tool for research into these processes. Additionally, fNIRS suitability for use in naturalistic settings may lead to the development of new research approaches with greater ecological validity compared to existing neuroimaging techniques.

## Introduction

The olfactory sense is responsible for the detection, encoding and perception of odours. Humans have an excellent sense of smell (Porter et al., 2006; Yeshurun and Sobel, 2010), and are reportedly able to discriminate more than one trillion olfactory stimuli (Bushdid et al., 2014). Despite these abilities, the human olfactory sense is underappreciated (Boesveldt and Parma, 2021), with one survey reporting that 53% of youths would rather give up their sense of smell than give up technology (McCann WorldGroup, 2011). The evolutionary decline of human olfactory use to allow for greater development of visual systems has even led some to consider olfaction as nothing more than a vestigial sense (Speed and Majid, 2018). These attitudes to olfaction are also mirrored in clinical settings. Unlike disorders of vision and hearing, olfactory disorders are not routinely screened for despite olfactory change or impairment being an early warning sign in many diseases including schizophrenia (Moberg, 1999; Turetsky et al., 2009; Kamath et al., 2017) and neurodegenerative conditions (Postuma et al., 2011; Doty, 2012; Hüttenbrink et al., 2013; Lucassen et al., 2016). Olfactory disorders have been associated with social isolation, poor mental and emotional health, decreased ability to detect and avoid environmental hazards, and an increased financial burden associated with funding treatment (Stevenson, 2009; Neuland et al., 2011; Croy et al., 2014; Erskine and Philpott, 2019). Odours and olfactory cues also influence health decision making, food choices and addiction maintenance behaviours (Tiggemann and Kemps, 2005; Patel et al., 2015; Kleinhans et al., 2020; Roose and Mulier, 2020; Sehrig et al., 2020). The olfactory sense also has a strong influence on emotion and wellbeing (Warrenburg, 2005). Different odours have been demonstrated to modulate mood, and feelings of stress and anxiety (Lehrner et al., 2000; Fukada et al., 2011; Kaimal et al., 2020). During and following the years of COVID-19 infection the impact to olfaction due to infection complications became a significant metric for long-COVID effects (Kapoor et al., 2021; Tan et al., 2022; Paranhos et al., 2023). Further research could advise applications of olfaction interventions in health and wellbeing.

As with olfaction, olfactory imagery can play a role in health-decision making and addiction maintenance behaviours. Olfactive imagery is the process of mentalising odours or olfactive experiences. As with other sensory modalities, forming a mental olfactory image has been shown to recruit sensory regions involved in olfactory perception (Djordjevic et al., 2005; Bensafi et al., 2007; Rinck et al., 2008). Along with visual and gustatory imagery, olfactory mental imagery forms a key component of food cravings (May et al., 2004; Tiggemann and Kemps, 2005). Olfactory imagery tasks have been shown to reduce food and cigarette cravings (Kemps and Tiggemann, 2007, 2009; Versland and Rosenberg, 2007). Guided mental imagery interventions using olfactory mental pictures have also been applied to improve health and wellbeing in clinical populations. It has been consistently demonstrated that olfactomotor activity during olfactory imagery mimics that of odour perception; olfactory imagery is associated with “sniffing” behaviours, as well as increased respiratory volume and depth (Bensafi et al., 2003, 2005; Arshamian et al., 2008; Kleemann et al., 2008). Forming pleasant olfactory mental imagery has also been shown to improve arterial oxygenation, and reduce the incidence and extent of atelectasis in patients following open heart surgery (Rezaei-Nodehi et al., 2018).

Despite many people reporting being able to generate olfactive images, debate still occurs as to whether olfactive imagery is a “true” form of imagery (Stevenson and Case, 2005). Whilst the mechanisms of other forms of mental imagery, such as visual imagery, are well documented, these do not seem to transfer across to imagery generation in the olfactory domain. As Stevenson and Case (2005) describe, formation of a visual mental image comprises the retrieval of an encoding from long-term memory, instantiation in the short-term visual store, and the representation of the encoding in a perceptual form. However, debate still occurs as to whether humans have an olfactory specific short-term or working memory capacity (White, 1998; Stevenson and Case, 2005). Evocation of olfactive imagery is often described as inconsistent and resource intensive, with generated images often being described as fleeting (Stevenson and Case, 2005; Plailly et al., 2011) and extremely vulnerable to confounding influences (Herz and von Clef, 2001; González et al., 2006; Royet et al., 2013a,b). However, olfactive imagery capacity has demonstrated a degree of plasticity, improving with frequency of use and expertise in the olfactive domain (Plailly et al., 2011; Royet et al., 2013a,b). Understanding the mechanisms of olfactory imagery could allow new approaches to access these health and wellbeing benefits associated with olfactory imagery.

One method that could be employed to reliably evoke olfactive imagery is to harness naturally occurring crossmodal interactions. A crossmodal interaction is where information from two individual sensory modalities, such as vision and smell, are integrated to create a sensory percept involving information from both modalities, known as a multimodal percept. The human brain is inherently geared towards multimodal sensory processing (Deroy and Spence, 2013); sensory stimuli are rarely experienced in one single modality. The crossmodal interaction between two stimuli can be driven by either semantic or synaesthetic congruence (Molholm, 2004; Hein et al., 2007; Spence, 2011). A strong cross-modal interaction occurs between olfactory and visual information (Gottfried and Dolan, 2003; Österbauer et al., 2005; Novak et al., 2015; Ripp et al., 2018; Sijben et al., 2018; Stickel et al., 2019). Visual information has been shown to aid in the detection, discrimination and labelling of odours (Gottfried and Dolan, 2003; Novak et al., 2015). The processing of visual stimuli has been demonstrated to exert a priming effect on secondary and tertiary olfactory regions (Gottfried and Dolan, 2003). As olfactory imagery also involves many secondary and tertiary olfactory regions (Djordjevic et al., 2005; Bensafi et al., 2007), these crossmodal correspondences can also be used to facilitate olfactory imagination. Understanding and employing these cross-modal interactions between visual and olfactory stimuli could aid in the research and applications of olfaction and olfactory imagery.

The olfactory sense has demonstrated extreme inter-individual variability (Morrot et al., 2012; Yunpeng et al., 2020); olfactory perceptual abilities can vary as a result of experience (Plailly et al., 2011; Royet et al., 2013a,b; Nováková et al., 2018), genetic factors (Keller et al., 2007; Josefsson et al., 2017), age (Doty et al., 1984; Mobley et al., 2014), gender (Royet et al., 2003), and contextual factors (Gottfried and Dolan, 2003; Herz, 2003; Laudien et al., 2008). As olfactory imagination abilities are highly correlated with olfactory perceptual abilities (Plailly et al., 2011; Royet et al., 2013a,b; Flohr et al., 2014; Kollndorfer et al., 2015a), it follows that olfactory imagery abilities are subject to these same confounding influences. Large variability in the olfactory sense poses a challenge to the generalisability of olfactory-based research. Similarly, reproducibility of olfactory findings is reliant on either stringent control of sample characteristics, which may threaten generalisability, or large sample sizes. However, high instrumentation running costs, and time requires for data acquisition and analysis, places a constraint on the participant sample sizes which can be analysed with current cognitive neuroscience research methodologies (Button et al., 2013; Szűcs and Ioannidis, 2017).

Current research into olfaction, olfactory imagery and crossmodal visual-olfactory interactions is also limited in ecological validity (Reader and Holmes, 2016; Elliott et al., 2021). Restrictive, unnatural environments required for electroencephalography (EEG) and functional magnetic resonance imaging (fMRI) are not conducive to natural olfactory perception and imagination processes. Poor motion tolerance can limit participants’ ability to interact with olfactory stimuli in a naturalistic manner. Methods of odour delivery must also be carefully designed to ensure instrumentation does not introduce noise or artefacts in neuroimaging data (Gorodisky et al., 2021). Neuroimaging environments, particularly the MRI scanner environment, have been demonstrated to impede perceptual decision-making and attentional focus (Van Maanen et al., 2015). Olfactory processing and imagination are cognitively demanding tasks which require a substantial degree of attentional focus (Zelano et al., 2004; Keller, 2011; Plailly et al., 2011; Royet et al., 2013a,b; Young, 2019). Due to the limitations in the ecological validity of current research methodologies in olfaction, olfactory imagery and crossmodal visual-olfactory processes, findings must be interpreted with caution; the gap between controlled experimental conditions and natural olfactory-based experiences may not allow neuroscientific research within these domains to translate into real-world contexts.

fNIRS is an emergent neuroimaging technique which can provide real-time insights into brain function during cognitive processes. Leveraging the unique capabilities of fNIRS could provide solutions to the current challenges in the research of olfaction, olfactory imagery and visual-olfactory interactions. fNIRS uses near-infrared (NIR) light to monitor changes in regional cerebral blood volume and hemodynamics. Light sources and detectors placed on the scalp direct NIR light at two discrete wavelengths into the brain, and the intensity of back-scattered light is recorded to monitor localised changes in oxygenated (HbO) and deoxygenated (HbR) haemoglobin (for further information, see Scholkmann et al., 2014). Compared to existing neuroimaging technologies, fNIRS is relatively cheap, easy to set up, and does not require a specialist environment (Pinti et al., 2020). The relative ease and lower costs of performing neuroimaging research using fNIRS can allow for data collection on a much wider scale than with fMRI. Applying fNIRS technology to the field of olfactory imagery research can allow data collection across a broader sample size to ensure the generalisability of research within these domains. Additionally, fNIRS exceptional motion tolerance has allowed for the application of wearable devices to conduct research in naturalistic settings (Pinti et al., 2018); using fNIRS could allow olfaction, olfactory imagery and visual-olfactory integration to be studied in naturalistic settings, producing more reliable and ecologically valid data.

However, fNIRS is limited in its recording depth; the channel between a source and detector pair interrogates the cerebral tissue between them at a maximum depth of roughly half the source-detector separation distance (Quaresima and Ferrari, 2019). The maximum source-detector separation that can be used to maintain a detectable signal is 3 cm, resulting in a recording depth of roughly 1.5 cm from the scalp surface. Olfaction is an evolutionarily old sense in humans, and as such, the functional centres associated with olfaction are mostly subcortical regions in the evolutionarily early areas of the brain such as the piriform cortex (PC), amygdala, insula and hippocampus (Royet et al., 2003; Djordjevic et al., 2005; Plailly et al., 2007; Han et al., 2019; Zhou et al., 2019). These regions are too deep for monitoring using fNIRS. However, several cortical regions have also been implicated in olfactory, imagery and crossmodal visual-olfactory processes such as the orbitofrontal cortex (OFC), middle and inferior frontal gyri (MFG, IFG) and inferior parietal lobe (IPL) (Plailly et al., 2011; Morrot et al., 2012; Meunier et al., 2014; Zhou et al., 2019; Iravani et al., 2021). These regions may be accessible for monitoring using fNIRS technology (see Discussion).

This review seeks to evaluate contemporary knowledge, debate and research themes in the fields of olfaction, olfactive imagery and crossmodal visual-olfactory correspondences. In particular, this review aims to identify key brain regions associated with these cognitive processes, and the common methodological approaches used, to determine whether neuroimaging with fNIRS would be a suitable tool for research into olfaction, olfactive imagery and crossmodal visual-olfactive correspondences. We have recently summarised and reviewed in Gunasekara et al. (2022), the current status of using fNIRS in olfaction. We now seek to expand this to other neuroimaging modalities and assess the use of neuroimaging approaches and paradigms within olfactive imagery and crossmodal visual-olfactory integration, and advise best practise when applying fNIRS technology in these domains.

## Methods

**Figure 1:**
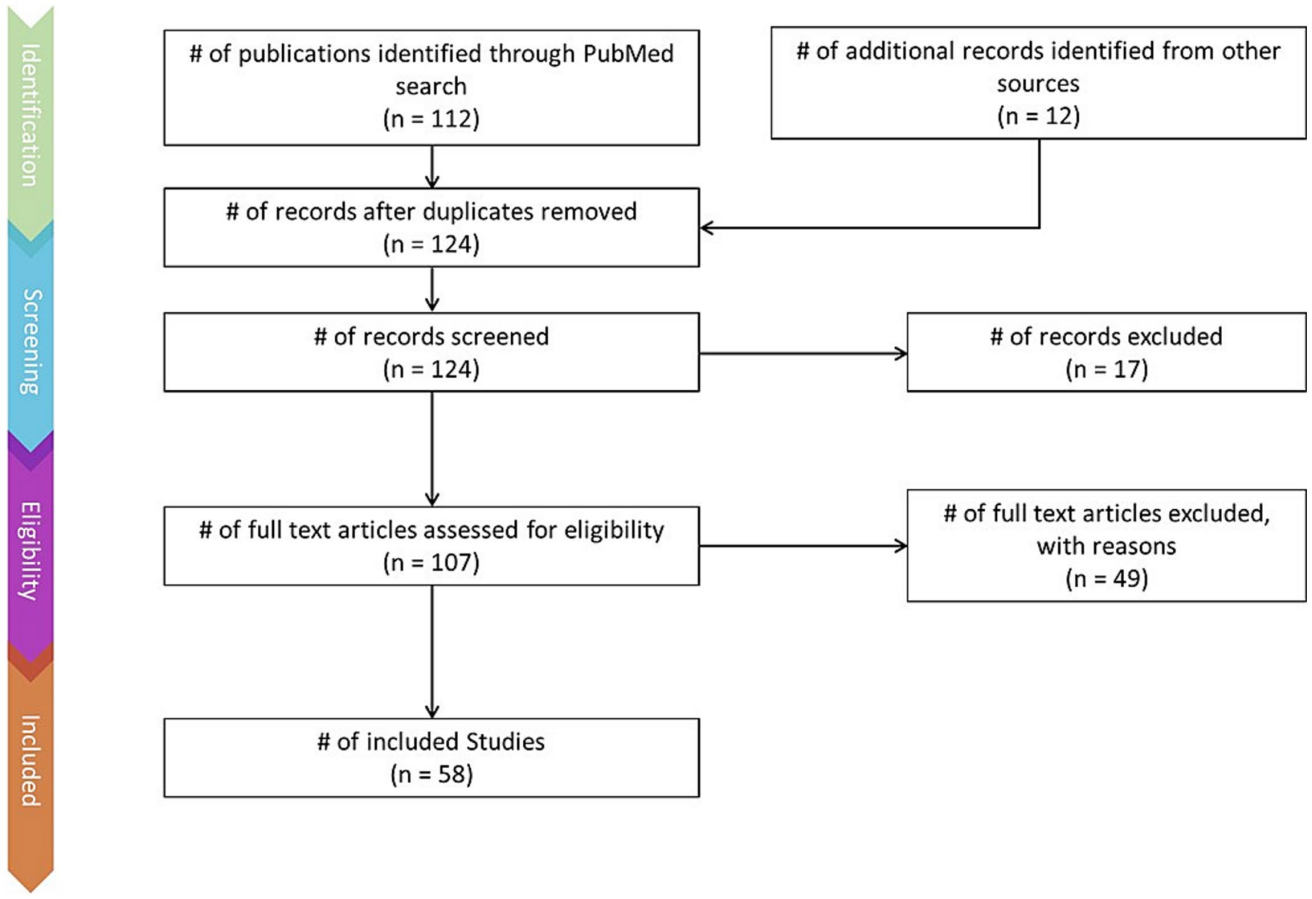
PRISMA flow-chart depicting the literature screening process, including number of articles found via keyword searches and additional sources, number of articles excluded, and number of articles retained.

This review was conducted using the Preferred Reporting Items for Systematic Reviews and Meta-Analyses (PRISMA) method (Page et al., 2021). The PRISMA flow chart (Figure 1) depicts the literature identification and screening process.

**Figure 2:**
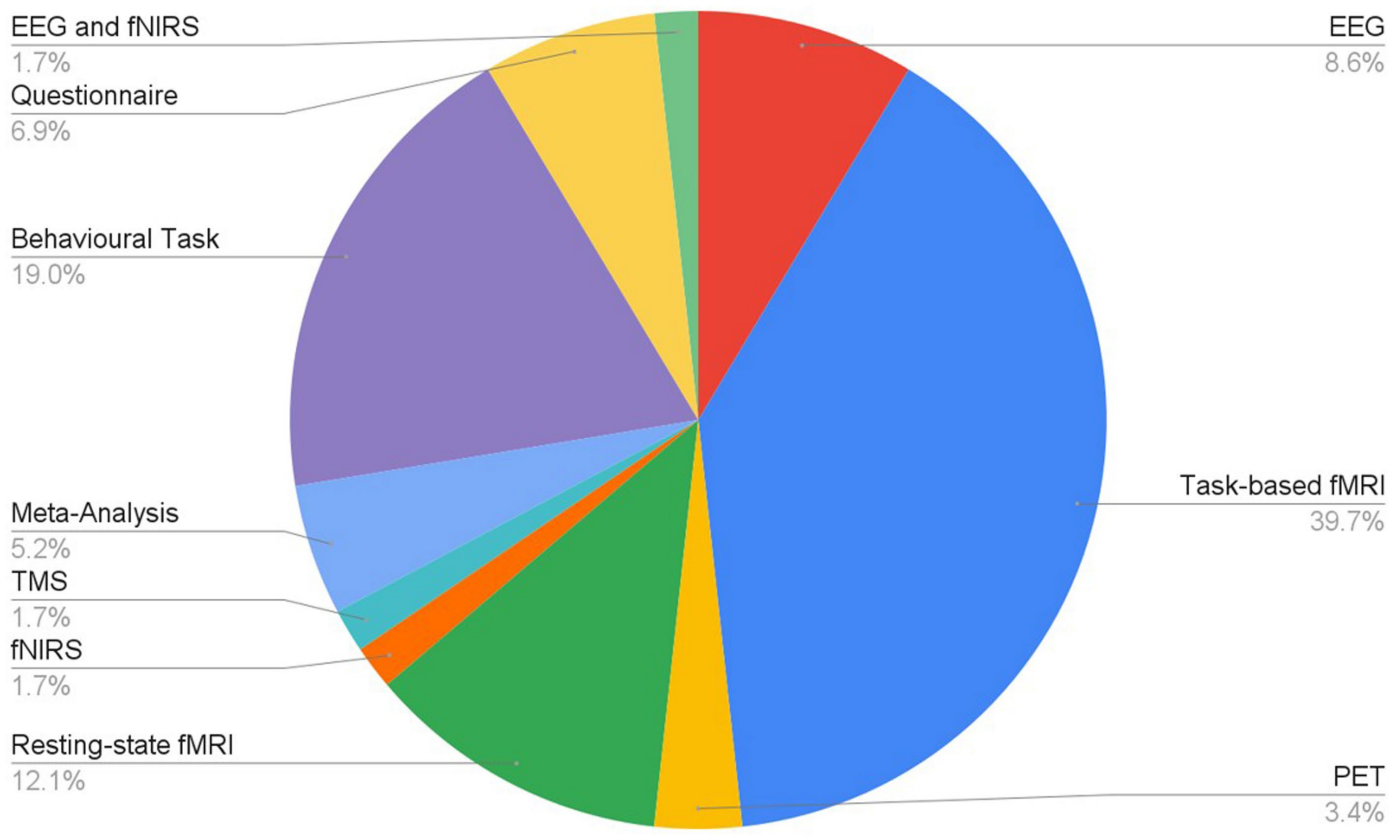
Distribution of research methodologies employed for research into olfaction, olfactive imagery and crossmodal interactions.

**Figure 3:**
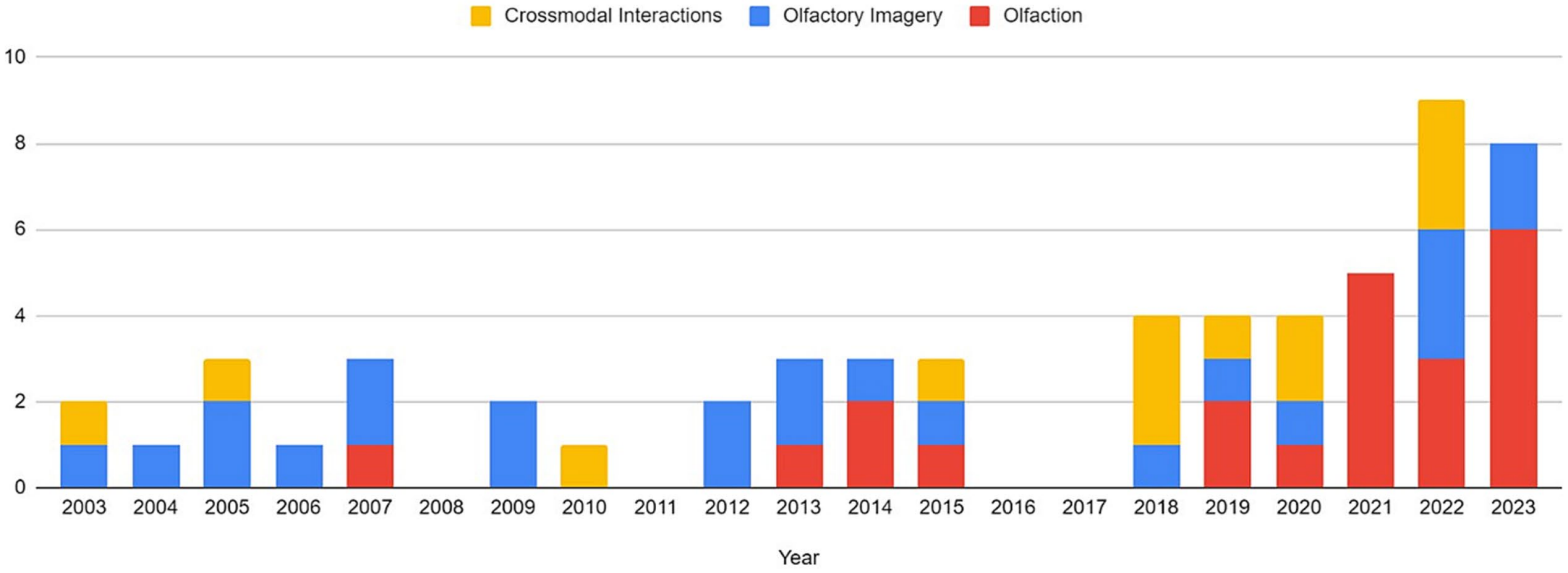
Distribution of publications related to olfaction, odour imagery and crossmodal visual-olfactory integration by year. For the purposes of this review, the search range was restricted to 2003–2023.

Articles were identified via a keyword search of the PubMed database using combinations of the key terms [odour|olfactory imagery|human olfaction|crossmodal|visual-olfactory|neuroimaging]. Boolean operators “AND,” “OR,” and “NOT” were used to combine key terms into search terms. Papers published between 2003 and 2023 were retained for review. Using these search terms, a total of 112 papers were identified through the PubMed database. For the purposes of this review, non-human studies and medical case reports were excluded. Additionally, papers referring to non-evoked olfactory experiences such as olfactory hallucinations or olfactive auras preceding migraines and seizures were excluded. Following screening for these criteria, 17 papers were excluded. Following review of full text articles, a further 49 papers were removed. Reasons for removal included irrelevance and incomplete method reporting. Review articles were retained or excluded on a case-by-case basis. Additionally, 12 articles were identified through other sources. This resulted in a total of 58 articles included in this review: 3 review articles and 55 primary research reports. Forty articles reported using neuroimaging research methodologies, 15 articles reported only using behavioural methodologies; 23 papers used task-based fMRI, 7 papers used resting state fMRI, 5 papers used EEG approaches, 2 papers used positron emission tomography (PET), one paper used transcranial magnetic stimulation (TMS), one paper used fNIRS, one paper used multimodal fNIRS and EEG, 11 papers employed behavioural task methods, 4 used questionnaires and 3 performed meta-analyses. Methods are summarised in Figure 2. A total of 36 studies used healthy, non-clinical participants, 19 used a clinical or specific population. These population groups included 6 using anosmic participants, 4 used participants with post-COVID-19 olfactory dysfunction, 1 using epileptic participants, 1 using blind participants, 1 used autistic participants, 2 contrasted student and expert perfumers, and 4 compared specific age groups. The distribution of reviewed publications per year is summarised in Figure 3; the number of publications has remained mostly consistent over the past 20 years. Publications regarding olfactory imagery have remained consistent between 2003 and 2023. Publications regarding crossmodal visual-olfactory interactions remained consistently low until 2018 which saw three publications on this topic, with interest continuing up to 2023. With network-based and connectivity approaches increasing in popularity, this increase in publications from 2018 may reflect an increasing interest to revisit the topic of crossmodal interactions using these approaches to characterise the network and connectivity characteristics which underlie visual-olfactory integrations. Of the six publications regarding crossmodal interactions published from 2018 onwards, three used connectivity-based analyses and two investigated network-based dynamics during visual-olfactory integration. Publications regarding olfaction dramatically increased in 2021 with five publications in one year followed by a further nine publications across 2022 and 2023. It is likely that this increased interest in olfaction research is associated with the coronavirus pandemic, with olfactory loss being a common symptom of COVID-19 infection. Seven of the fourteen olfactory publications from 2021 to 2023 compared normosmic with dysosmic or anosmic participants. With olfactory dysfunction remaining a prevalent symptom of COVID-19 and long-covid, it is likely that olfactory research will continue to see increased interest over the next few years (Table 1).

**Table 1:** Summarising articles reviewed on the topics of olfaction, olfactive imagery and crossmodal olfactory-visual integration.

References	Cognitive process	Participants	Method	Protocol	Conclusions
Amsellem et al. (2018)	Visual-olfactory interactions and subjective experience	14 participants	Behavioural tasks	Unimodal or bimodal visual and olfactory stimulation	Visual and olfactory stimuli are processed in parallel, interactions influence various levels of subjective experience.
Arnold et al. (2020)	Human olfactory network organisation	728 participants	Resting-state fMRI	Scanned at rest	Identified olfactory functional network and provided network-level insights into functional specialisation and spatial segregation of the olfactory system.
Arshamian et al. (2020)	Different sensory embodiment effects on imagery across modalities	61 adults, 120 children	Questionnaire	PSI-Q	Olfactory imagery does not become more vivid with age and is different to representations from other senses.
Bensafi and Rouby (2007)	Olfactory and emotional perception abilities impact on odour imagery	40 participants	Questionnaire	VVIQ, VOIQ, PAS, ETOC	Olfactory imagery is related to emotion and good and bad imagers differ in experience of emotions and long term memory of smells.
Bensafi et al. (2005)	Sniffing patterns during odour imagery	Exp 1: 10 participants Exp 2: 30 participants Exp 3: 40 participants	Behavioural tasks	Form auditory, olfactory or visual mental images	Sniffing behaviours facilitate odour imagery and may serve as a reliable tool for exploring individual differences in odour imagery.
Bensafi et al. (2007)	Hedonic specific piriform activity in olfaction and odour imagery	14 participants	Task-based fMRI	Smell or imagine odours following a preparatory cue.	Evidence of activation of primary sensory olfactory regions during olfactory imagery.
Callara et al. (2021)	Hedonic olfactory perception	30 participants	Task-based EEG	Presented with odours of different valence	Interactions with the OFC and brain regions associated with emotion recognition and memory dynamically change with odour valence.
Djordjevic et al. (2004)	Effects of odour and visual imagery on odour detection	72 participants	Behavioural task	Odour, visual or no mental imagery followed by an odour detection task	Effect of imagery on detection is content- and modality-specific.
Djordjevic et al. (2005)	Odour imagery compared with odour perception	67 behavioural screening, 12 retained for scanning	Task-based PET	Smell or imagine odours following a preparatory cue	Neural networks engaged in odour perception and odour imagery partially overlap.
Douaud et al. (2022)	Brain functional and structural changes following COVID-19 infection	401 post-covid, 384 control	Resting-state fMRI	Scanned at rest	Covid-19 infection associated with degeneration of olfactory regions and pathways, and cognitive decline.
Eek et al. (2023)	Passive smelling, odour encoding and odour recognition	25 participants	Task-based fMRI	Three odour stimulation tasks to target passive smelling, odour encoding and odour recognition	Identified regions associated with lower- and higher-order olfactory functions
Fallon et al. (2020)	Effect of visual congruence on olfactory habituation	Exp 1: 25 participants Exp 2: 25 participants	Task-based EEG	Prolonged floral odour exposure during presentation of congruent or incongruent visual stimuli	Congruent visual stimuli enhances olfactory sensitivity to prolonged odour stimulation
Flohr et al. (2014)	Odour imagery following olfactory loss	16 anosmic, 19 healthy control	Task-based fMRI	Imagine odours then rate mental image	Olfactory loss is associated with difficulties performing olfactory imagery in the conventional way, and regular exposure to olfactory information could help maintain imagery capacity.
González et al. (2006)	Word induced olfactory brain responses	23 participants	Task-based fMRI	Reading olfactive vs control words	Reading olfactory words is associated with activation or language and olfactory areas.
Gorodisky et al. (2021)	Odour induced brain activity and valence of odours	20 normosmic, 2 anosmic	Task-based fMRI	Passive odour perception with novel odour canopy method	Using novel odour canopy method generates typical olfactory response in the brain.
Gottfried and Dolan (2003)	Crossmodal visual facilitation or olfactory perception	17 participants	Task-based fMRI	Unimodal vs bimodal odour detection task	Human hippocampus mediates reactivation of crossmodal semantic associations, even in the absence of memory processing.
Han et al. (2019)	Human olfactory dysfunction	19 studies	Meta-analysis	Review of brain regions associated with olfactory dysfunction	Summarises structural and functional alterations associated with olfactory loss and regain and new approaches for future clinical practise.
Han et al. (2022)	Effect of generating odour imagery in individuals with low olfactory imagery abilities	49 participants	Task-based fMRI	Imagine odours in a long vs short imagery period	When generating odour images in a shorter time period, high and low ability odour imagers may adopt different approaches.
Hörberg et al. (2020)	Visual dominance in visual-olfactory multisensory integration	30 participants	Task-based ERP	Bimodal object categorisation with competing olfactory and visual stimuli	Contrary to the idea of visual dominance, incongruent odours may uniquely attract mental processing resources during perceptual incongruence.
Hucke et al. (2023)	Neural spatial representations of odour locations	Exp 1: 18 participants Exp 2: 14 participants	Task-based EEG and fNRIS	Monorhinal odour stimulation presented at different intensities	Trigeminal odour stimulation is required to create spatial representation of odour presentation.
Hudry et al. (2014)	Lateralisation of olfactory processing in patients with temporal lobe epilepsy	28 right TLE, 33 left TLE, 60 control	Behavioural task	Odour perception, rating and naming	Global olfactory impairments in TLE and evidence for lateralised olfactory processing.
Infortuna et al. (2022)	Motor cortex responses to pleasant odour perception and imagery, impact of personality and imagery abilities	25 participants	Task-based TMS and EMG	Changes in rMT and MEP amplitude during odour perception and imagery.	Perception and imagination of odours modulates motor cortex excitability providing evidence for interactions between olfactory and motor systems.
Iravani et al. (2021)	Functional connectivity and morphology in acquired olfactory loss	20 anosmic, 23 healthy control	Resting-state fMRI	Scanned at rest	Recent sensory loss is associated with changes in core olfactory areas and increased dynamic functional connectivity from olfactory regions to multisensory integration regions.
Kärnekull et al. (2021)	Verbally induced olfactory illusions and visual influence	17 early blind, 15 late blind, 32 sighted	Behavioural task	Odours presented with negative, neutral and positive labels.	General mechanisms underlying verbally induced olfactory illusions are not caused by visual processing or visual mental imagery.
Kleemann et al. (2008)	Breathing parameters during odour perception and olfactory imagery	56 participants	Behavioural task	Odour perception followed by mental recall of odour	Olfactory perception and imagery both have effects on respiratory profile based on a common underlying mechanism.
Kollndorfer et al. (2015a)	Ability to self-evaluate olfaction and imagery abilities	43 anosmic, 16 hyposmic and 16 healthy control	Questionnaires	Sniffin' sticks, self reported sense of smell (1 to 9), VOIQ	Participants who were able to perceive odours rely on the vividness of their mental odour images to evaluate their olfactory performance.
Kollndorfer et al. (2015b)	Olfactory training in long term anosmia	19 healthy control, 10 anosmic, 7 anosmic followed up	Task-based fMRI	Odour intensity rating before and after 12 week olfactory training	Olfactory training can reorganise functional networks although no differences in spatial distribution were observed.
Kretzmer and Mennemeier (2022)	Hemispheric integration in olfactory stimulation	44 participants	Behavioural task	Olfactory bilateral vs unilateral stimulation with ratio scaling response	Findings consistent with a summation model of olfactory integration across left and right hemispheres.
Leclerc et al. (2019)	Olfactory imagery source memory	48 participants	Task-based fMRI	Smell or imagine odours, or hear or imagine words.	Olfactory imagery is susceptible to source memory errors, and distinct neural networks underlie auditory and olfactory imagery involving different areas of the SMA.
Martial et al. (2023)	Passive odour perception and alertness	21 participants, all male	Resting-state fMRI	Lemon or no odour presented and alertness rated	Higher alertness after lemon inhalation versus rest and increased network integration in olfactory regions
McNorgan (2012)	Multisensory and modality specific imagery	65 research reports	Meta-analysis	ALE and MKDA techniques.	Modality-specific imagery regions overlap but are not confined to somatosensory and motor execution areas. The is also a general imagery network recruited regardless of task.
Meunier et al. (2014)	Olfactory memory networks	16 young, 22 elderly	Task-based fMRI	Identification of old vs new odours.	Neural networks involved in odour recognition memory are organised into modules and the modular partitions are linked to behavioural performance.
Morrot et al. (2012)	Individual variability in olfactory regions	76 participants	Task-based fMRI	Odour or visual stimuli detection task.	Low reliability of olfactory activations means fMRI is not a suitable diagnostic tool for neurodegenerative disease in single subjects.
Muccioli et al. (2023)	Cognitive and functional connectivity impairment in post-COVID-19 olfactory dysfunction	19 hyposmia, 26 control	Resting-state fMRI	Scanned at rest	Persistent OD following COVID-19 is associated with altered olfactory network connectivity which correlates with severity.
Novak et al. (2015)	Subthreshold negative emotion perception from olfactory-visual integration	16 participants	Task-based fMRI	Rating of valence in odours and sub-threshold emotional faces.	Findings confirm involvement of multisensory convergence areas and unique areas in olfaction-related integration and support inverse effectiveness principle.
Österbauer et al. (2005)	Odour responses in human brain with co-occurring colour stimuli	9 participants	Task-based fMRI	Unimodal or bimodal visual and olfactory stimulation.	Neuronal correlates of olfactory response are modulated by colour cues in brain areas previously associated with hedonic value of odours.
Palmiero et al. (2013)	Imaginative vs semantic processing	87 participants	Behavioural task	Two experiments comparing imaginative and semantic processing in vision, audition and olfaction.	Visual and auditory imaginative processing can be differentiated from semantic processing, though imagery relies heavily on semantic representations.
Perszyk et al. (2023)	Odour imagery, perception and food cue reactivity	45 participants	Task-based fMRI	Oodur perception and imagination task.	Accuracy of decoding imagined but not real odour quality correlated with odour imagery ability and greater adiposity mediated by cue-potentiated craving and food intake.
Plailly et al. (2007)	Odour discrimination	16 participants	Task-based PET	Odour detection and odour discrimination task.	Successively discriminating between odours activates a left lateralised frontotemporal network involving olfactory regions and working memory regions.
Plailly et al. (2011)	Functional reorganisation of brain regions involved in odour imagery in experts	14 student and 14 expert perfumers	Task-based fMRI	Odour imagery task and passive odour perception task.	Olfactory expertise is associated with a functional reorganisation of olfactory and memory brain regions allowing increased ability to imagine odours and create fragrances.
Raj et al. (2023)	Cognitive influence on odour identification errors in age related smell loss	2479 older adults	Behavioural	Odour naming task from a set of target and distractor names	Odour identification errors are partially explained by semantic and perceptual similarities.
Rekow et al. (2022)	Crossmodal olfactory facilitation in visual categorisation	26 participants	Task-based EEG	Ambiguous and unambiguous visual stimuli presented with or without a congruent odour	Congruent body odour facilitate rapid, automatic visual categorisation of ambiguous face stimuli.
Ripp et al. (2018)	Multisensory olfactory-visual integration	18 participants	Task-based fMRI	Unimodal or bimodal visual and olfactory stimulation.	Identified a multisensory integration processing specific network involved in olfactory-visual integration.
Royet et al. (2003)	Emotional responses to odours	28 participants	Task-based fMRI	Pleasant and unpleasant odour perception.	Lateralised processing of odours varies with handedness and gender. Left hemisphere is involved in judgements of odour pleasantness.
Royet et al. (2013a,b)	Odour mental imagery in non-experts	14 student and 14 expert perfumers	Task-based fMRI	Reanalysis of data from Plailly et al. (2011)	Evidence of odour imagery capabilities in non-experts, however the neurophysiological and cognitive processes vary with expertise.
Schicker et al. (2022)	Removing a modality during visual-olfactory stimulation	20 middle aged, 13 older adults	Task-based fMRI	Unimodal vs bimodal visual olfactory stimulation with removal of a modality at the end of bimodal trials	Removal of a modality from a bimodal presentation results in additional brain activity associated with attention, memory, and searching for the missing stimulus.
Schienle et al. (2017)	Emotion-specific nocebo effects	29 participants, all female	Task-based fMRI	Affective image task whilst wearing odourless patch under nose.	Nocebo elicited an aversive odour response to visually induced disgust, and modulated OFC activation and connectivity.
Schlintl et al. (2022)	Olfactory imagery for autobiographical memory retrieval	296 participants, all female	Behavioural	Asked to generate non-specific odour mental imagery	Odour imagery more effective than visual imagery in retrieving unpleasant adulthood memories or pleasant childhood memories but evoked less diverse emotions.
Seo et al. (2010)	Cross-modal integration between odours and abstract symbols	Exp 1: 120 participants Exp 2: 42 participants	Task-based EEG	Pleasant or unpleasant odour presented with congruent, incongruent or no abstract shapes	Congruent shapes increased pleasantness and unpleasantness ratings of odours and modulated N1 amplitude and latency. Evidence of abstract shapes modulating odour perceptual experience.
Sijben et al. (2018)	Semantic congruence in olfactory-visual perception	19 participants	Task-based fMRI	Congruent, semi congruent or incongruent visual and olfactory stimuli.	Identified left IFG involvement in multisensory integration across different congruence levels which would not have been possible with a subtractive design.
Stickel et al. (2019)	Audio-visual and olfactory-visual integration in autistic vs healthy controls	18 autistic and 17 healthy controls	Task-based fMRI	Unimodal vs bimodal olfactory-visual or audio-visual stimuli.	Multisensory integration has shared neural sources across olfactory-visual and audio-visual stimulation in patients and controls. Enhanced recruitment of the IPS modulates changes between areas relevant to sensory perception.
Tomasino et al. (2022)	Multisensory mental imagery following covid-19	55 with olfactory or gustatory dysfunction, 20 without following Covid-19	Questionnaire	PSI-Q, VOIQ and two custom questionnaires.	COVID-19 infection frequently causes hyposmia and dysgeusia, and may also alter mental representations responsible for olfactory and gustatory perception.
Tomiczek and Stevenson (2009)	Effects of odour naming on imagery ability	31 participants, all female	Behavioural task	Repetition priming and recognition naming task	Trying to form an odour image facilitates performance by producing a generic state of activation, which only benefits existing odour-name associations.
Torske et al. (2022)	Functional anatomy of the olfactory system	81 research reports	Meta-analysis	ALE technique	Identified olfactory brain areas with significant peaks across all reviewed brain areas, and regions specific to different odour categories.
Wingrove et al. (2023)	Olfactory network functional connectivity in post-COVID-19 OD	57 participants, grouped based on antibody and chemosensory status	Resting-state fMRI	Scanned at rest	Identifies functional differences in olfactory, sensory processing and cognitive functional areas associated with post-COVID OD
Yamashita et al. (2022)	Harmony between colours and odours	5 participants	Task-based fNIRS	Participants smelled odours in synaesthetically or semantically congruent or incongruent coloured lighting	Synaesthetic-driven crossmodal interactions are more congruent than semantic-driven
Yunpeng et al. (2020)	Individual differences in olfactory brain activations in normosmia/dysosmia	22 dysosmic, 16 normosmic	Task-based fMRI	Presented with alternating blocks of coffee smell or odourless air	Large inter-individual variabilities for odour-induced brain activation means it appears problematic to diagnose olfactory dysfunction on an individual level using fMRI.
Zhou et al. (2019)	Functional pathways in human olfactory system	25 participants	Resting-state fMRI	At rest, breathing through nose	Results provide insight into the functional and anatomical organisation of the human olfactory system.

ALE, activation likelihood estimate; EEG, electroencephalography; EMG, electromyography; ETOC, European Test of Olfactory Capabilities; fMRI, functional magnetic resonance imaging; fNIRS, functional near-infrared spectroscopy; MEP, motor-evoked potential; MKDA, multilevel kernel density analysis; PAS, physical anhedonia scale; PET, positron emission tomography; PSI-Q, Plymouth sensory inventory questionnaire; VOIQ, vividness of olfactory imagery questionnaire; VVIQ, vividness of visual imagery questionnaire.

### Olfaction

**Figure 4:**
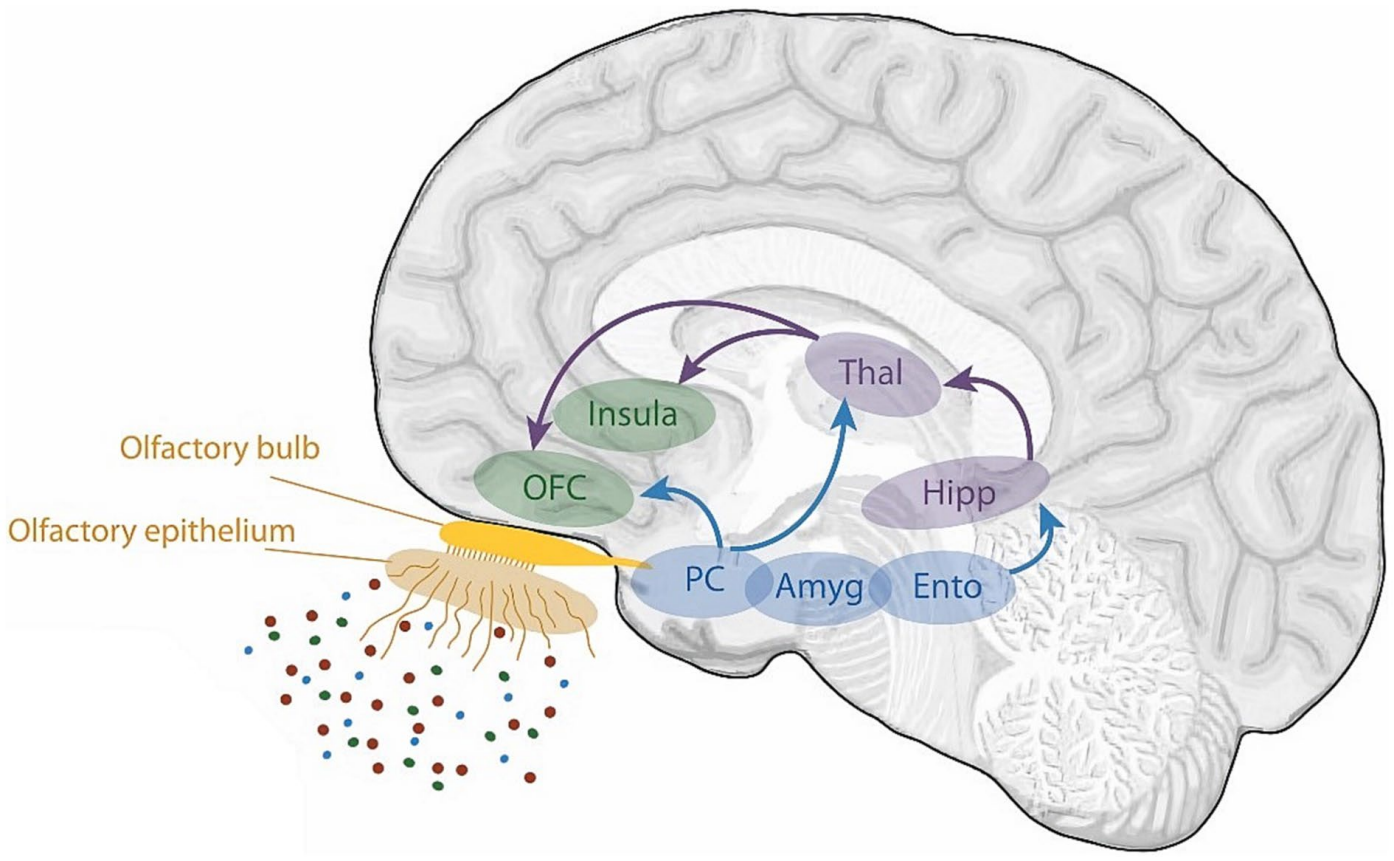
A schematic view of the human olfactory system. The primary and secondary olfactory regions are represented in blue and green, respectively. Amy, amygdala; Ento, entorhinal cortex; Hipp, hippocampus; OFC, orbitofrontal cortex; PC, piriform cortex; Thal, thalamus. Retrieved from Saive et al. (2014).

**Figure 5:**
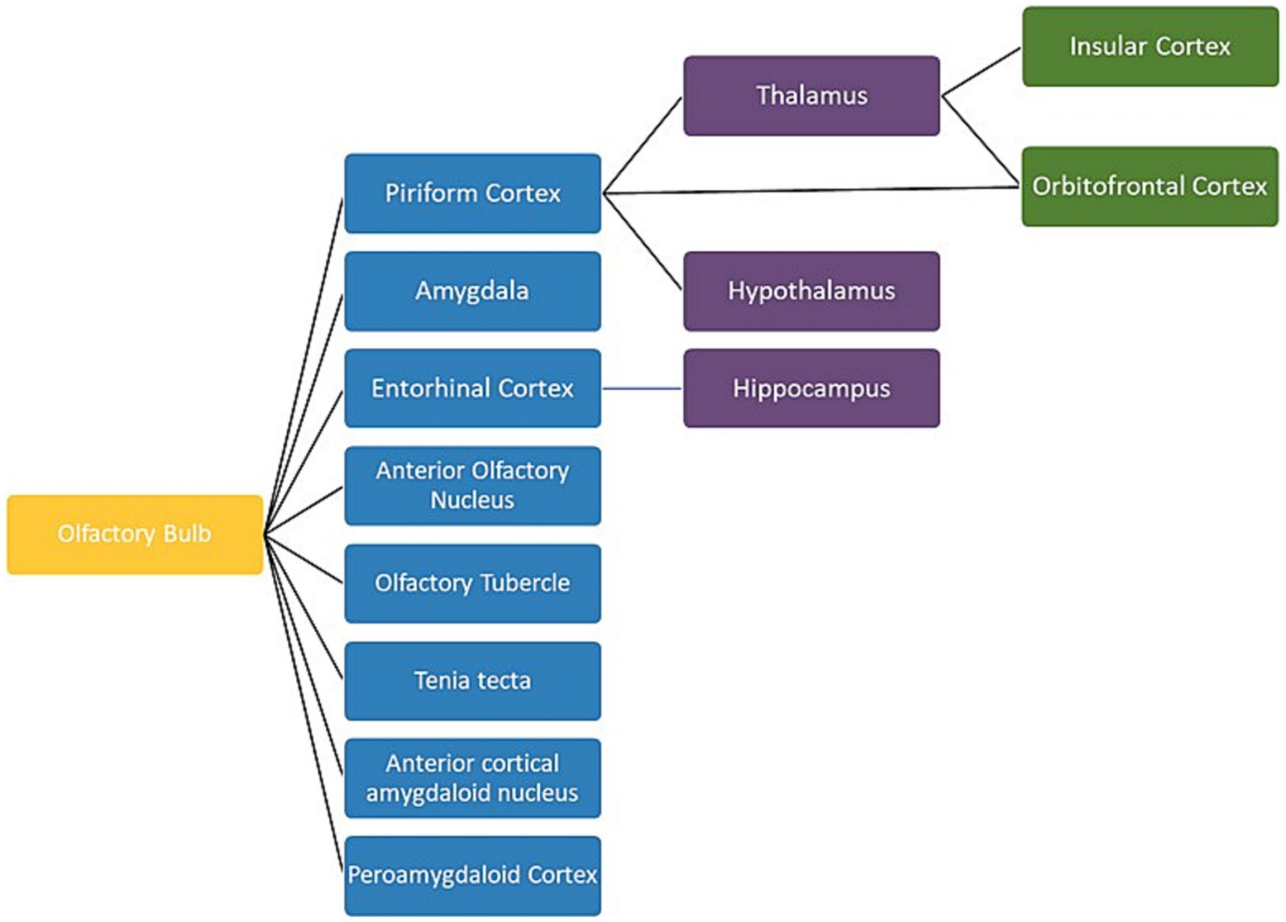
A schematic view of the human olfactory system. The primary, secondary and tertiary olfactory regions are represented in blue, purple and green respectively.

**Figure 6:**
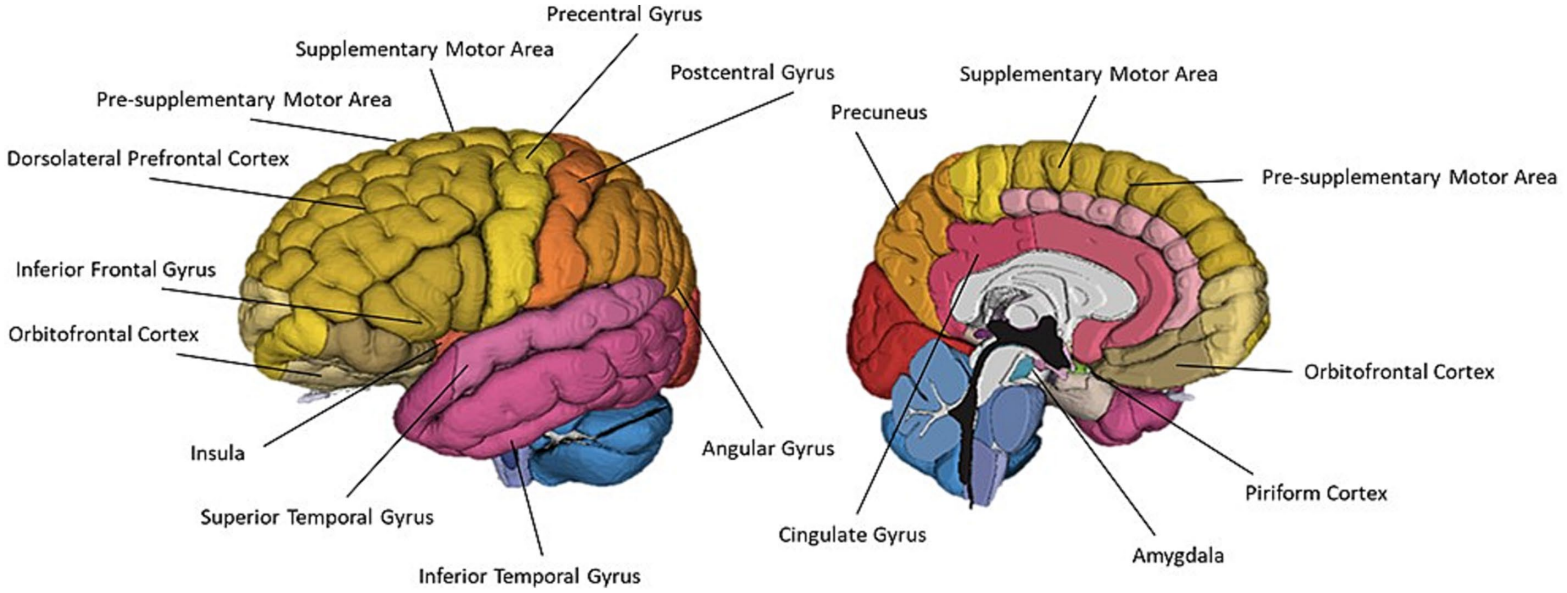
A schematic representation of commonly cited regions involved in olfaction as identified in this review.

Thirty-two of the reviewed articles studied aspects of olfaction; 22 employed neuroimaging techniques, eight used behavioural methods and two performed meta-analyses. fMRI was the most used neuroimaging method, with ten neuroimaging papers using a task-based fMRI method and seven using resting-state fMRI. The bias for employment of fMRI methodology, which has exceptional spatial resolution, reflects the common research theme of localising olfactory processes within the brain. As the primary and secondary olfactory regions have been extensively documented prior to 2002 (see Figures 4, 5), many of the reviewed papers instead seek to localise specific higher level cognitive olfactive processes. The regions associated with different olfactory-related cognitive processes are summarised in Table 2 and Figure 6.

**Table 2:** Summarising key regions associated with olfaction.

		Subcortical			Frontal						Parietal			Temporal	
References	Cognitive process of interest	PC	Amyg	CgG	Insula	OFC	DLPFC	IFG	SMA/preSMA	PrCG	PostCG	PrC	AG	STG	ITG
Arnold et al. (2020)	Human olfactory network organisation	x	x		x	x									
Bensafi et al. (2007)	Hedonic specific piriform activity in olfaction and odour imagery	x			x	x									
Callara et al. (2021)	Hedonic olfactory perception			x		x						x		x	
Djordjevic et al. (2005)	Odour imagery compared with odour perception	x		x	x	x	x		x						
Douaud et al. (2022)	Brain functional and structural changes following COVID-19 infection	x	x	x	x	x									
Eek et al. (2023)	Passive smelling, odour encoding and odour recognition	x	x	x	x		x					x			
Gorodisky et al. (2021)	Odour induced brain activity and valence of odours	x	x			x									
Han et al. (2019)	Human olfactory dysfunction	x	x	x	x	x									
Hucke et al. (2023)	Neural spatial representations of odour locations		x	x		x				x	x				
Iravani et al. (2021)	Functional connectivity and morphology in acquired olfactory loss	x						x				x	x		x
Kollndorfer et al. (2015b)	Olfactory training in long term anosmia			x	x				x	x		x			
Martial et al. (2023)	Passive odour perception and alertness									x			x	x	
Meunier et al. (2014)	Olfactory memory networks	x		x	x		x	x				x			
Morrot et al. (2012)	Individual variability in olfactory regions	x	x	x		x	x	x							
Muccioli et al. (2023)	Cognitive and functional connectivity impairment in post-COVID-19 olfactory dysfunction		x	x	x	x									
Perszyk et al. (2023)	Odour imagery, perception and food cue reactivity	x	x	x	x	x	x		x	x	x				
Plailly et al. (2007)	Odour discrimination		x		x	x		x		x				x	
Plailly et al. (2011)	Functional reorganisation of regions involved in odour imagery in experts	x	x						x			x			
Royet et al. (2003)	Emotional responses to odours	x	x	x	x	x		x		x				x	
Torske et al. (2022)	Functional anatomy of the olfactory system	x	x	x	x	x	x	x		x	x				
Wingrove et al. (2023)	Olfactory network functional connectivity in post-COVID-19 OD			x	x	x									
Yunpeng et al. (2020)	Individual differences in olfactory brain activations in normosmia/dysnomia	x			x	x									
Zhou et al. (2019)	Functional pathways in human olfactory system	x		x	x	x		x	x	x	x		x		x

Cortical areas which may be suitable for monitoring using fNIRS are shaded grey (see Discussion). PC, piriform cortex; Amyg, amygdala; CgG, Cingulate gyrus; OFC, orbitofrontal cortex; DLPFC, dorsolateral prefrontal cortex; IFG, Inferior frontal gyrus; SMA, supplementary motor area; pre-SMA, pre-supplementary motor area; PrCG, precentral gyrus; PostCG, postcentral gyrus; PrC, precuneus; AG, angular gyrus; STG, Superior temporal gyrus; ITG, Inferior temporal gyrus.

Another common theme involved localisation of olfactory function and functional changes within specific populations. Eight studies evaluated participants with olfactory dysfunction, and other studied population groups included student vs. expert perfumers, younger and older participants, early and late blind participants, and participants with temporal lobe epilepsy.

With olfactory regions well established, another common approach was to characterise the involvement of these regions within the wider network. The increasing popularity of functional network analyses within cognitive neuroscience is allowing characterisation of localised brain region function in cognitive processes which are lost in subtractive models. Ten of the reviewed studies employed a functional connectivity or network-based analysis approach, and it is likely that olfactory research will continue to see an increase in this approach, as is seen in other cognitive neuroscience domains.

A prominent research theme across the reviewed articles was the study of hedonics; ten studies considered the impact of odour valence on olfactory processing. Seven of these articles directly contrasted odour valence: two behavioural (Hudry et al., 2014; Kärnekull et al., 2021) and five neuroimaging (Royet et al., 2003; Bensafi et al., 2007; Callara et al., 2021; Gorodisky et al., 2021; Torske et al., 2022). A further three papers involved discussion of the impact of odour hedonicity on olfactory processes, but did not directly manipulate odour pleasantness (Bensafi and Rouby, 2007; Plailly et al., 2007; Morrot et al., 2012). Royet et al. (2003) further extended this by exploring the impact of handedness, gender and active hedonic judgements on hedonic odour processing. The lateral aspect of the left OFC was implicated in mediating the conscious assessment of odour pleasantness, with this lateralisation being particularly pronounced in female participants. All seven of the neuroimaging papers which included themes of hedonic odour processing also cited OFC involvement. Other regions commonly cited for their involvement in hedonic odour processing included the piriform cortex, cingulate gyrus (CgG), superior temporal gyrus (STG), amygdala and insula.

Royet et al. (2003) identification of differential involvement of the left and right OFC in olfactory processing also supports evidence of the lateralisation of olfactory processing. First proposed by Broman et al. (2001), the differential involvement of the left and right hemispheres in olfactory processing remains a pertinent topic of discussion within olfactory research. Broman et al. suggested the right hemisphere is involved in low-level perceptually based odour processing and encoding, and the left hemisphere is associated with higher-level cognitive-based odour recognition processes and semantic interpretation. Royet et al. findings support this theory, with the left OFC expressing greater involvement that the right OFC in the judgement of odour pleasantness. Nine other reviewed articles discussed or presented evidence to support this lateralisation in olfactory processing (Royet et al., 2003; Djordjevic et al., 2005; Bensafi et al., 2007; Plailly et al., 2007; Hudry et al., 2014; Kollndorfer et al., 2015b; Zhou et al., 2019; Douaud et al., 2022; Kretzmer and Mennemeier, 2022; Eek et al., 2023).

Hudry et al. (2014) provided further evidence of left hemisphere dominance in semantic and emotional olfactory processing by studying participants with unilateral temporal lobe epilepsy (TLE). Their findings highlighted the privileged role of the left hemisphere for emotional and semantic processing, with left TLE participants judging odours as less pleasant and exhibiting greater difficulty with identification. Furthermore, the reported advantage for judging odour familiarity during right nostril stimulation validates the role of the right hemisphere in encoding the sensory percept of an odour; familiarity ratings largely reflect the clarity of perceptual processing (Broman et al., 2001; Royet, 2004).

Kollndorfer et al., 2015b evaluated three functional networks involved in olfactory processing labelled as the olfactory network, the somatosensory network, and the integrative network. They reported the olfactory network was relatively symmetrical across both hemispheres, whereas the somatosensory network expressed significantly greater right hemisphere recruitment and the integrative expressed a clear left hemisphere bias. Zhou et al. (2019) performed a laterality index analysis to quantify functional asymmetry of the olfactory processing. Similarly to Kollndorfer et al., they did not identify any significant asymmetry across the primary olfactory network. These findings, suggest that odours are perceived equally by both hemispheres, but that each hemisphere proceeds to encode different aspects of the odour: the right hemisphere encoding the olfactory perceptual experience, and the left hemisphere encoding emotional and semantic interpretations of the odour.

The most notable theme within the reviewed olfactory research was the characterisation of olfactory memory. Fourteen of the reviewed papers weighed in on the debate regarding olfactory memory processes. Whilst only three of the reviewed papers actively studied olfactory memory (Plailly et al., 2007; Meunier et al., 2014; Eek et al., 2023), eleven papers were able to apply their findings to contribute further knowledge to the discussion of olfactory memory (Djordjevic et al., 2004; Bensafi and Rouby, 2007; Plailly et al., 2011; Hudry et al., 2014; Kollndorfer et al., 2015a; Zhou et al., 2019; Callara et al., 2021; Infortuna et al., 2022; Torske et al., 2022; Muccioli et al., 2023; Perszyk et al., 2023). Memory of odours and olfactory experiences presents a unique case of memory encoding and recall compared to other sensory modalities (Stevenson and Case, 2005; White, 2009; Eek et al., 2023). As such, it is understandable that there is great interest in the study of olfactory memory, and it remains such a prominent topic of research within olfactory research.

Olfactory memories are highly resistant to forgetting over time and often experienced with higher emotional intensity (Roediger et al., 2017). As shown by Callara et al. (2021) and Zhou et al. (2021), the olfactory system has connections with both the amygdala and hippocampus. Whilst other sensory systems must relay through the thalamus (Eek et al., 2023), the olfactory system is directly communicating with centres associated with emotion and memory. Callara et al. (2021) identified the OFC as the node with the highest inflow during olfactory stimulation, noting its key role in olfactory perception. They also identified strong interactions between the OFC and brain regions associated with emotion and memory. They conclude that these connections may be responsible for the enhanced encoding and emotional intensity of olfactory memory.

Discourse surrounding odour memory is inherently associated with the lateralisation of olfactory processing. The same left-right dichotomy in odour processing appears to be mirrored within odour memory encoding and recall (Broman et al., 2001; Royet, 2004; Hudry et al., 2014). The “dual process theory” describes two memory processes contributing to stimulus recognition: “familiarity,” described as perceptual recognition of an odour related to implicit or unconscious memory, and “recollection,” described as conceptually driven recognition along with contextual information retrieval involving explicit or conscious memory (Royet, 2004; Eek et al., 2023). These memory processes are associated with the right and left hemispheres respectively, mirroring the described laterality of olfactory processing (Royet, 2004; Hudry et al., 2014). Hudry et al. (2014) study particularly highlights the complex interplay between the hemispheres in the recollection and familiarity of odours; odour identification was impaired in participants with left TLE, whereas odour familiarity ratings were associated with a clear right-nostril advantage.

Another point of discussion within olfactory memory research pertains to the existence of an olfactory working memory capacity (White, 1998; Stevenson and Case, 2005). The reviewed literature presents a general consensus to support the existence of a working memory. One method to interrogate olfactory working-memory is through odour discrimination; discrimination between successive odour stimuli requires working memory involvement to hold the perceptual trace of the first stimuli for comparison with the subsequent odour presentation. Plailly et al. (2007) employed an odour discrimination paradigm inspired by the *n*-back task, a common paradigm used to investigate working memory, to evaluate olfactory working-memory. Authors identified activations in the left IFG and OFC associated with the maintenance of the first odour perceptual trace, demonstrating the existence of an olfactory working-memory capacity.

Working-memory capacity in the olfactory domain can also be investigated via tasks which require the maintenance of a neural representation of olfactory stimuli. For example, Hucke et al. (2023) used a combined EEG and fNIRS methodology to investigate the requirement of trigeminal stimulation for neural representation of odour source localisation. The involvement of somatosensory cortices during localisation of odour stimuli indicates the dorsal network involvement in processing where a stimulus occurs, as has been extensively documented during visual processing, also extends to olfactory processing (Frasnelli et al., 2012; Hucke et al., 2023). These results also support the sensorimotor recruitment models of working memory whereby the systems involved in the sensory perception of stimuli can also hold a short-term representation of sensory information (D’Esposito and Postle, 2015). This provides further support for the existence of an olfactory working-memory capacity which mirrors that of other sensory modalities.

The prominence of memory discussion in olfactory processing also appears to be closely related to the research theme of odour hedonics, as hedonic judgements are mainly driven by memory and semantic smell identification (Schleidt et al., 1988; Sucker et al., 2007). The performance of hedonic odour judgement, particularly of unpleasant odours was consistently associated with left hemisphere involvement within the reviewed literature. Given the evidence surrounding the lateralisation of odour memory, it appears that this left hemisphere bias is indicative of sematic and contextually driven odour recollection processes, mediated by the left hemisphere.

All the reviewed neuroimaging literature reported activation within at least one of the documented olfactory processing regions. The reviewed literature presents a consensus as to the lateralisation of olfactory function, with the right hemisphere associated with low-level olfactory perceptual processing, and the left hemisphere associated with higher level cognitive olfactory processing including hedonic judgements, odour naming, semantic interpretation and olfactory memory. The three most prominent themes within the reviewed literature; hedonic odour perception, lateralisation of odour processing and olfactory memory, all appear to be very closely related. Hedonic odour perception was associated with mostly left-lateralised regions including left OFC, CgG, STG, piriform, and amygdala, and bilateral insulae. Multiple studies reported activation in regions associated with memory recall and working memory, including the PrC, SPL and IFG. The reviewed studies appear to provide support for the existence of an olfactory-specific working-memory capacity. This supports the notion that olfactory imagery is mediated by the same mechanisms underlying other imagery modalities, and hence is a “true” form of sensory imagery.

### Olfactory imagery

**Figure 7:**
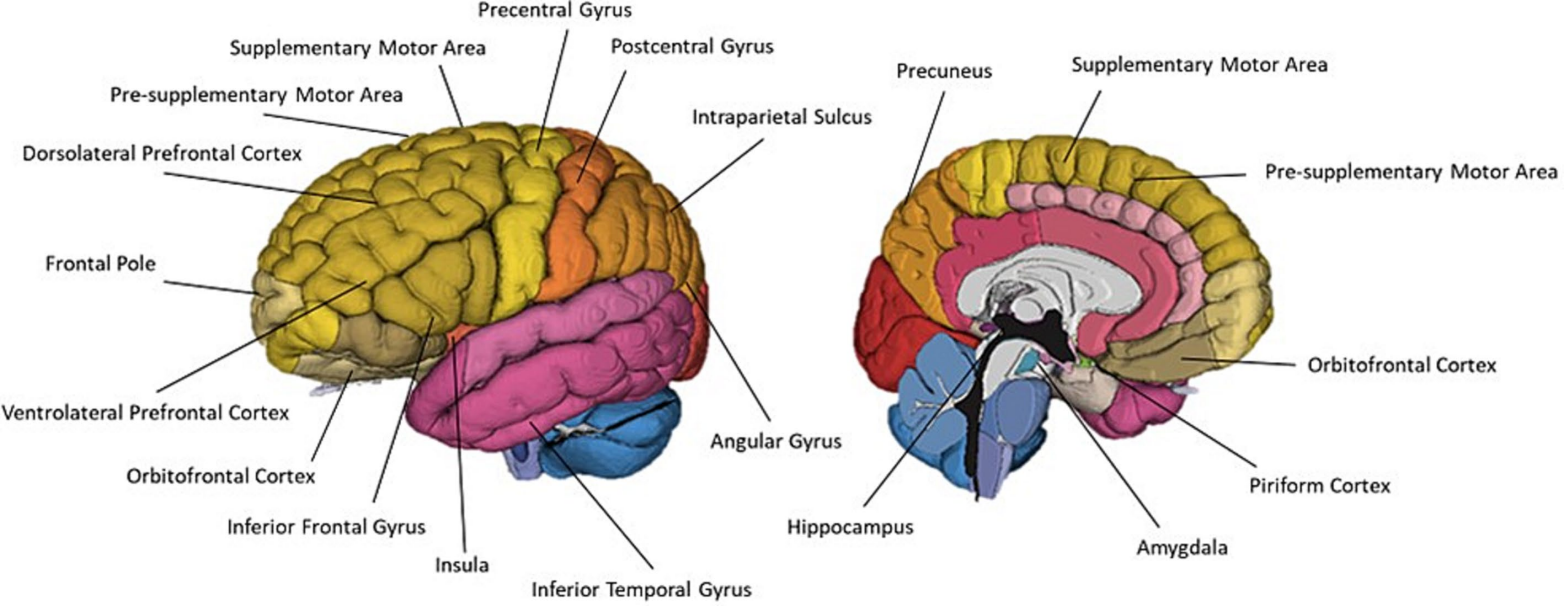
A schematic representation of commonly cited regions associated with olfactory imagery as identified in this review.

Twenty-two articles studied olfactive imagery. Eleven papers employed neuroimaging methods, ten employed behavioural methods and one performed a meta-analytic review. Nine neuroimaging papers used task-based fMRI, one used PET and one used TMS and EMG. Once again, the dominance of fMRI in olfactory imagery research appears to reflect a common aim of localising olfactory imagery regions within the brain. A summary of brain regions associated with olfactory imagery is presented in Table 3 and Figure 7.

**Table 3:** Summarising key regions associated with olfactory imagery.

		Subcortical			Frontal								Parietal				Temporal
References	Cognitive process of interest	PC	Hippo	Amyg	Insula	FP	OFC	SFG	MFG/DLPFC	IFG/VLPFC	SMA/preSMA	PrCG	PostCG	PrC	IPS	AG	ITG
Bensafi et al. (2007)	Hedonic specific piriform activity in olfaction and odour imagery	x			x		x										
Djordjevic et al. (2005)	Odour imagery compared with odour perception	x			x		x		x		x				x		
Flohr et al. (2014)	Odour imagery following olfactory loss	x	x	x	x		x		x					x			
González et al. (2006)	Word induced olfactory brain responses	x		x	x					x							
Han et al. (2022)	Effect of generating odour imagery in individuals with low olfactory imagery abilities		x					x		x	x			x			x
Leclerc et al. (2019)	Olfactory imagery source memory					x	x		x	x	x				x	x	
McNorgan (2012)	Multisensory and modality specific imagery		x	x	x										x		
Perszyk et al. (2023)	Odour imagery, perception and food cue reactivity	x			x				x	x	x	x					
Plailly et al. (2011)	Functional reorganisation of brain regions involved in odour imagery in experts	x	x	x	x	x	x	x	x			x		x			x
Royet et al. (2013a,b)	Odour mental imagery in non-experts	x	x	x	x		x	x	x				x	x			x
Schienle et al. (2017)	Emotion-specific nocebo effects	x	x		x		x										

Cortical areas which may be suitable for monitoring using fNIRS are shaded grey (see Discussion). PC, piriform cortex; Amyg, amygdala; FP, Frontal pole; OFC, orbitofrontal cortex; SFG, superior frontal gyrus; MFG, middle frontal gyrus; DLPFC, dorsolateral prefrontal cortex; IFG, Inferior frontal gyrus; VLPFC, ventrolateral prefrontal cortex; SMA, supplementary motor area; pre-SMA, pre-supplementary motor area; PrCG, precentral gyrus; PostCG, postcentral gyrus; PrC, precuneus; IPS, Intraparietal sulcus; AG, angular gyrus; ITG, Inferior temporal gyrus.

Five neuroimaging papers sought to localise regions associated with olfactory imagery by contrasting imagery and perception (Djordjevic et al., 2005; Bensafi et al., 2007; Plailly et al., 2011; Leclerc et al., 2019; Perszyk et al., 2023). Leclerc et al. (2019) identified a largely left lateralised network including left DLPFC, IFG, IPS, angular gyrus and pre-SMA, and right frontal pole and IFG which was more active during odour imagery than during odour perception. This appears to mirror the prominent discourse around the lateralisation of olfactory processes within olfaction research; the findings of a mostly left-lateralised network associated with olfactory imagery further corroborates the left hemisphere is involvement in the higher level cognitive olfactory processes including odour memory and semantic labelling. Somewhat conversely, Djordjevic et al. (2005) compared olfactory perception and olfactory imagery, finding odour imagery efficiency scores were significantly correlated with rCBF increases in right anterior and posterior OFC. The authors concluded that this positive correlation suggests successful odour imagery occurs when the brain treats odour images the same as perceived odours.

**Figure 8:**
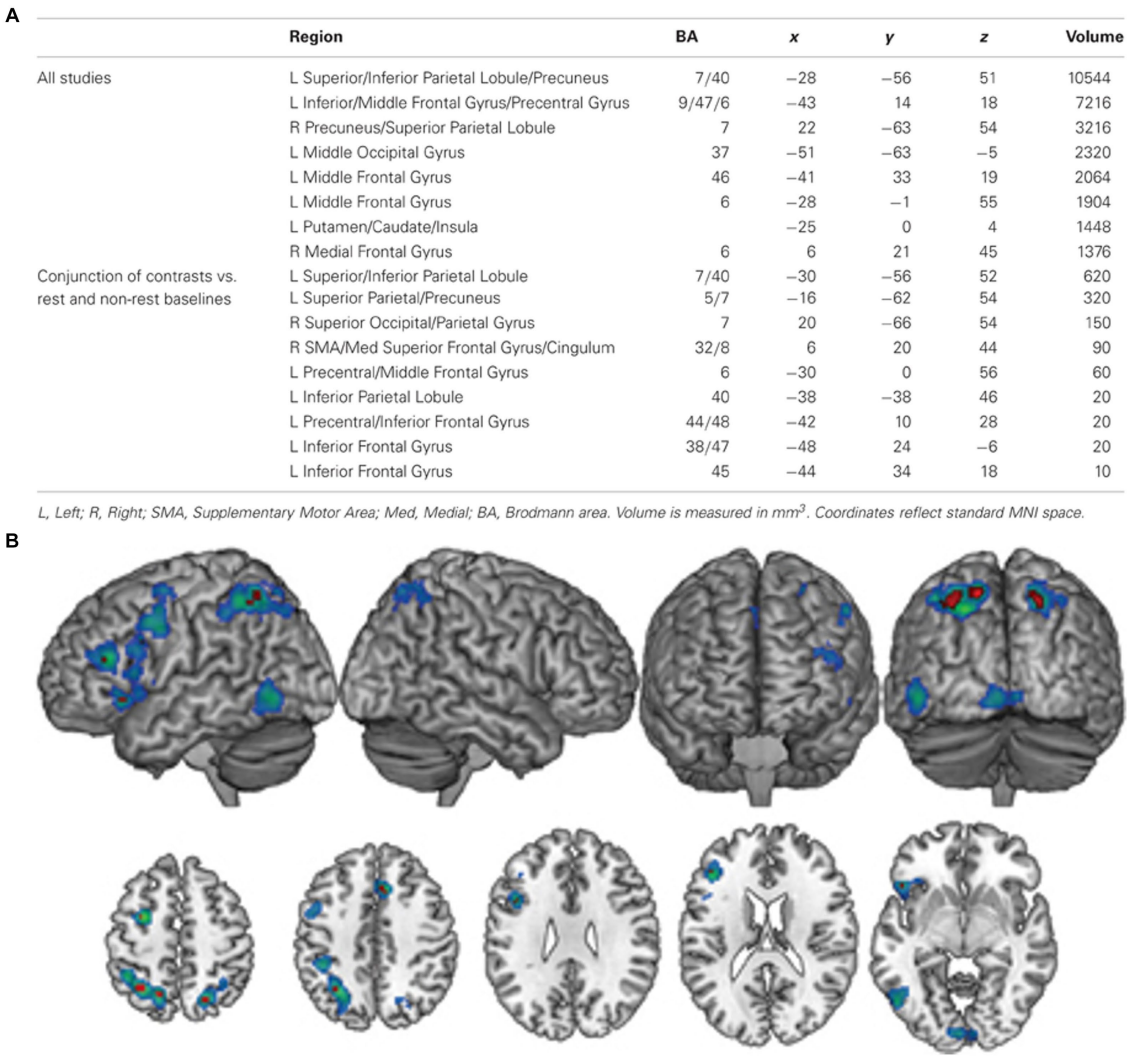
**(A)** A table of modality general regions identified by McNorgan (2012). **(B)** The general imagery network (cool colours) identified using ALE analysis. Conjunction analysis of studies comparing complex and resting-state baseline conditions identified nine clusters (hot colours) that were active across all imagery conditions, regardless of baseline task. L, left; R, right; SMA, supplementary motor area; Med, medial; BA, Brodmann area. Retrieved from McNorgan (2012).

Another approach to localise olfactory imagery regions contrasted olfactive imagery with imagery in other sensory modalities such as visual or auditory imagery. Four papers contrasted sensory specific imagery with modality general imagery regions and identified the left lateralised imagery network is modality general, but that there are also regions associated with olfactory-specific imagery (McNorgan, 2012; Flohr et al., 2014; Leclerc et al., 2019; Han et al., 2022). McNorgan (2012) performed a meta-analysis of articles studying uni- and multi-sensory imagery to localise imagery general and modality specific brain regions. They analysed 65 research reports across olfaction, audition, gustatory, motor, tactile, visual-colour, visual-form and visual-motion. Analysis identified a general imagery network of eight, mostly left lateralised regions. Four left-lateralised clusters exclusively associated with olfaction were identified in the anterior cingulate, hippocampus, amygdala and SPL. Similarly, Han et al. (2022) identified olfactory imagery was associated with greater activation in bilateral PrC (SPL) and superior occipital cortices, left hippocampus and right SFG than visual imagery. The authors concluded this increased involvement of the PrC, superior occipital regions (cuneus) and hippocampus in the odour imagery condition suggest that odour imagery may rely more on memory retrieval processes than visual imagery (Figure 8).

Most of the reviewed papers evaluated olfactory imagery in a healthy, non-clinical population. Due to the wide heterogeneity in olfactory imagery abilities across the general population, it is understandable that many reviewed studies still seek to characterise olfactory imagery in the general population, rather than focusing on subgroups with potentially atypical olfactory imagery. However, six papers did investigate specific populations. Studies which included specific populations included dysosmic or anosmic participants (Flohr et al., 2014; Kollndorfer et al., 2015a; Tomasino et al., 2022), young vs adult participants (Arshamian et al., 2020) and student vs expert perfumers (Plailly et al., 2011; Royet et al., 2013a,b). A common research theme within these papers was to consider the impact of olfactory exposure on olfactory imagery abilities. All the reviewed papers agreed that varying expertise was associated with the recruitment of different brain regions for olfactory imagination.

One potential reason for this difference in regions may be due to differences in imagery generation techniques. Han et al. (2022) unique paradigm investigated differences odour imagery generation across varying olfactory imagery abilities by employing short vs. long odour imagery generation times. Participants with lower olfactory imagery expressed stronger activation in the left SMA and right SFG in the short olfactory imagery condition than in the long olfactory imagery condition, brain regions involved in modality general mental imagery (McNorgan, 2012; Zvyagintsev et al., 2013). This increased activation of multisensory regions may indicate participants with lower olfactory imagery abilities formed mental images including other sensory modalities to facilitate olfactory imagination.

Another possible reason for these differences may be differences in retrieval effort of olfactory memories. Plailly et al. (2011) identified a bilateral network of regions including the right MFG which expressed reduced imagery-induced activation with expertise. They concluded that the activation decrease associated with increased olfactory imagery performance are reflective of the “retrieval effort” (Tulving, 1985); student perfumers at the beginning of their career must deploy a greater level of processing resources to retrieve the olfactive image than expert perfumers. These findings are further extended by Flohr et al. (2014) who identified increasing activation in bilateral DLPFC (MFG) associated with olfactory loss, and that the degree of DLPFC activation varies with longevity of olfactory dysfunction. Flohr et al. hypothesise this varying recruitment of DLPFC is the result of greater recruitment of working memory resources based on similar observations amongst the visually impaired (Dulin et al., 2011), also providing further evidence of an olfactory working memory capacity, and supporting the conclusion of Plailly et al. (2011) that increasing activation within these regions is indicative of greater retrieval effort correlating with lower olfactory expertise.

Royet et al. (2013a,b) re-analysed Plailly et al. (2011) data to identify changes to functional coactivation between 22 ROIs identified by Plailly et al., hypothesising that increasing olfactory expertise would be associated with greater connection across olfactory memory regions. They identified professional perfumers demonstrated significantly greater coactivations between MFG and the rest of the olfactory imagery network, and significantly lower coactivation between the PrC and rest of the imagery network than student perfumers. They concluded these changes to connectivity reflect differences in the recall mechanisms underlying olfactory imagery between student and expert perfumers. According to “multiple trace theory” of memory consolidation, retrieval-related activation of the hippocampus reduces over time, with more involvement of prefrontal cortex regions in the recall of more mature memories; retrieval of some distant memories can have no hippocampal involvement. The increased connectivity of the middle frontal gyrus with olfactory and memory regions in the expert group is likely indicative of post hippocampal memory recall in the expert group. In contrast, the increased coactivation of the PrC with memory and olfactory regions in the student group is indicative of allocation of top-down attentional resources to memory retrieval. Involvement of the superior parietal lobe, including the PrC, during memory recall has also been associated with lower confidence in the accuracy of mental imagery (Ciaramelli et al., 2008).

A key finding across the reviewed literature is the involvement of key memory retrieval and working memory regions in olfactory imagery. This supports the argument that olfactory imagery is a true form of sensory imagery. The involvement of memory regions is also demonstrated to vary with varying levels of olfactory expertise. It is hypothesised that this reflects differences in the mechanisms of retrieval, and use of polymodal imagery to facilitate odour imagery generation. Localisation of olfactory imagery regions compared to olfactory perception reveals a largely left lateralised olfactory imagery network including left DLPFC, IFG, IPS, angular gyrus and pre-SMA. This reflects the lateralisation of olfactory function as proposed by Broman et al. (2001) and prominently discussed within olfactory research that the right hemisphere is associated with the sensory perception of odours, and the left hemisphere is involved in the higher level cognitive olfactory processes including odour memory and semantic labelling. However, many of the regions identified in this network appear to be modality-general imagery regions. When contrasted with imagery in other sensory modalities, olfactory specific activity is observed in the anterior cingulate, hippocampus, amygdala and SPL, regions which have also been implicated in memory recall. It is likely that the enhanced involvement of memory recall regions within olfactory imagery when compared to other modalities is the result of greater retrieval effort required to form an olfactory mental image, and top-down attention direction towards the intended modality within an involuntary polymodal mental image formed to facilitate olfactory imagery generation.

### Crossmodal interactions

**Figure 9:**
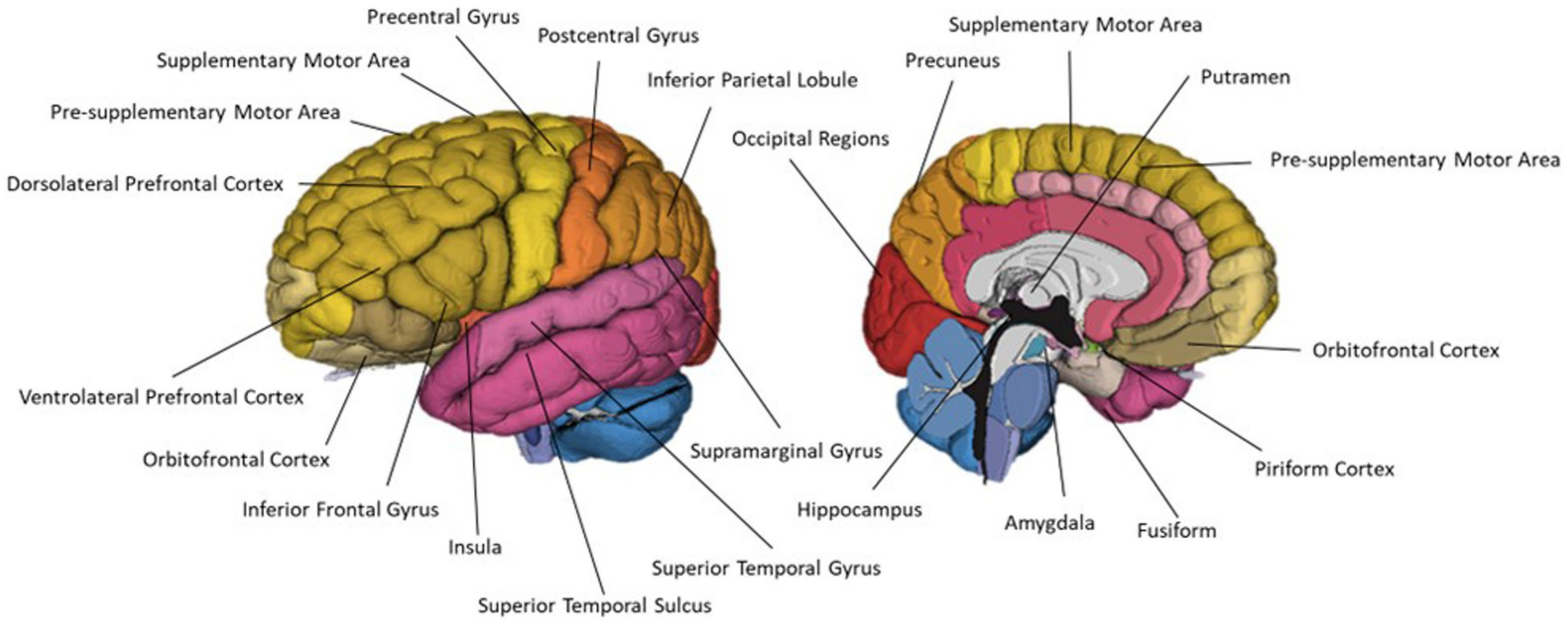
A schematic representation of commonly cited regions associated with crossmodal visual-olfactory integration identified in this review.

Thirteen studies investigated crossmodal interactions between vision and olfaction. Twelve of these papers employed neuroimaging techniques and one used only behavioural measures. The most common imaging modality was fMRI, employed by seven of the reviewed papers. This is likely reflective of a strong research aim of characterising the regions involved in crossmodal interactions. One study used fNIRS to investigate crossmodal colour-odour correspondances. The use of fNIRS in this study allowed the investigation of colour-odour correspondances using a unique paradigm which has not been used in previous neuroimaging investigation of crossmodal visual-odour correspondances. Regions associated with crossmodal interactions are summarised in Table 4 and Figure 9.

**Table 4:** Summarising key regions associated with crossmodal visual-olfactory integration.

		Subcortical					Frontal					Parietal				Temporal	Occipital	
References	Cognitive process of interest	PC	Insula	Hippo	Amyg	Put	OFC	MFG/DLPFC	IFG/VLPFC	SMA/preSMA	PrCG	PostCG	PrC	IPL	SMG	STG/STS	Occ	Fus
Gottfried and Dolan (2003)	Crossmodal visual facilitation or olfactory perception	x	x	x			x							x		x		
Novak et al. (2015)	Subthreshold negative emotion perception from olfactory-visual integration	x		x	x		x									x		
Österbauer et al. (2005)	Odour responses in human brain with co-occurring colour stimuli	x	x	x			x		x							x		
Ripp et al. (2018)	Multisensory olfactory-visual integration		x			x			x		x	x	x		x	x	x	x
Schicker et al. (2022)	Removing a modality during visual-olfactory stimulation								x		x	x					x	x
Sijben et al. (2018)	Semantic congruence in olfactory-visual perception		x			x		x	x						x	x		
Stickel et al. (2019)	Audio-visual and olfactory-visual integration in autistic vs healthy controls		x	x	x			x	x	x	x		x				x	x

Cortical areas which may be suitable for monitoring using fNIRS are shaded grey (see Discussion). PC, piriform cortex; Hippo, hippocampus; Amyg, amygdala; Put, putamen; OFC, orbitofrontal cortex; MFG, middle frontal gyrus; DLPFC, dorsolateral prefrontal cortex; IFG, Inferior frontal gyrus; VLPFC, ventrolateral prefrontal cortex; SMA, supplementary motor area; pre-SMA, pre-supplementary motor area; PrCG, precentral gyrus; PostCG, postcentral gyrus; PrC, precuneus; IPL, Inferior parietal lobule; SMG, supramarginal gyrus; STG, superior temporal gyrus; STS, Superior temporal sulcus; Occ, occipital regions; Fus, fusiform.

The most commonly used paradigm involved presenting participants with unimodal vs bimodal visual and olfactory stimuli. This protocol was used by six studies, five fMRI studies (Gottfried and Dolan, 2003; Österbauer et al., 2005; Ripp et al., 2018; Stickel et al., 2019; Schicker et al., 2022) and one behavioural study (Amsellem et al., 2018). Within the bimodal condition, all of these studies presented the bimodal stimuli as congruent or incongruent pairs. Additionally, Amsellem et al. (2018) included semi-congruent and semi-incongruent conditions. They achieved this by selecting two target odours and creating three additional blended fragrances with varying ratios of the two target odours. Through this, they were able to demonstrate that congruence between visual and olfactory stimuli is not a dichotomy, but rather that participants were able to detect the nuances of varying degrees of congruence, which impacted upon pleasantness ratings.

**Figure 10:**
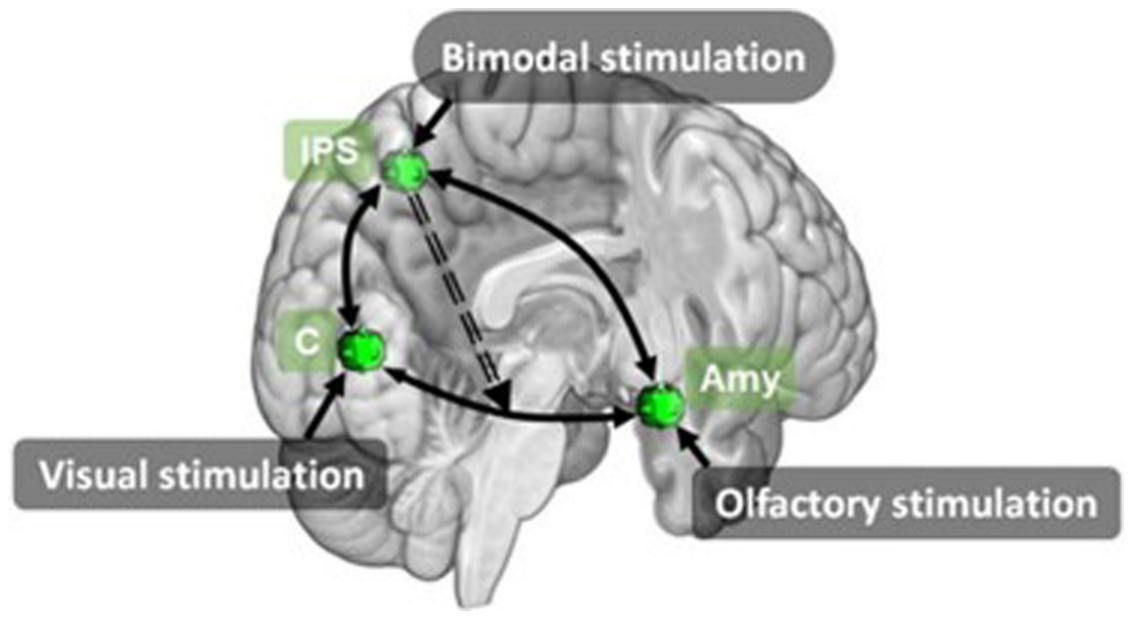
Effective connectivity model for olfactory-visual stimulation identified using DCM by Stickel et al. (2019). Amy, amygdala; C, cuneus; IPS, inferior parietal sulcus. Retrieved from Stickel et al. (2019).

In addition to unimodal vs bimodal conditions, three papers (Gottfried and Dolan, 2003; Ripp et al., 2018; Stickel et al., 2019) also included pleasant and unpleasant valence conditions. This resulted in four bimodal, four unimodal and one baseline condition. From this Gottfried and Dolan (2003) contrasted these conditions to identify brain regions associated with olfaction, pleasant and unpleasant odour perception, olfactory-visual interactions and congruence of olfactory-visual stimuli. Both Ripp et al. (2018) and Stickel et al. (2019) performed connectivity analyses. Stickel et al. (2019) investigated multisensory integration using DCM to analyse information exchange during bimodal olfactory-visual or auditory-visual stimulation. Using three key regions identified from the unimodal visual (cuneus), unimodal olfactory (amygdala) and bimodal congruent (IPS) conditions, Stickel et al. modelled the network linked to integration of visual and olfactory stimuli. Their model composed of a driving input of bimodal olfactory-visual stimulation to the IPS and nonlinear modulations from IPS to the reciprocal cuneus ↔ amygdala connection (Figure 10). Their results showed an overlapping network of brain regions involved in multisensory integration of olfactory-visual and audio-visual information. They also demonstrate the IPS modulates changes between areas relevant to sensory multisensory perception by exerting top-down control over primary sensory regions.

Ripp et al. (2018) also used a unimodal vs bimodal paradigm with congruent and incongruent, and pleasant and unpleasant conditions, and a graph theoretical network based functional connectivity analysis. Ripp et al. (2018) identified six nodes which expressed significantly stronger functional connectivity in the bimodal condition than the combination of unimodal conditions. Bimodal presentation of odour and pictures, collapsed across valence, was associated with significantly greater functional connectivity between the right putamen ↔ right insula, PrC ↔ left SMG and left MOG ↔ left IFG. Involvement of the right insula and putamen has been observed in previous studies of multisensory integration, regardless of sensory modality (Banati, 2000; Bushara et al., 2001; Olson et al., 2002; Naghavi et al., 2007; Renier et al., 2009), leading the authors to conclude that this connectivity between the insula and right putamen is part of a functional multisensory integration specific network. Involvement of the PrC, as cited in Royet et al. (2013a,b), is likely indicative of top-down facilitation of memory retrieval. Ripp et al. proposed that the increased connectivity between the PrC and SMG is indicative of memory retrieval and maintenance of the retrieved memory within a phonological store, once again supporting the evidence for an olfactory working memory. The left IFG has also been shown to be associated with odour working memory, semantic interpretation and odour naming (Djordjevic et al., 2005; Plailly et al., 2007). Ripp et al. suggested that the increased connectivity of the left MOG, a visual processing region, and left IFG allows the matching of visual information with odour semantic information. They further propose that retrieved odour memory information, held in the phonological store, along with visual information and semantic information from the left MOG and IFG are passed via the inferior fronto-occipital fasciculus, a large white matter tract connecting the frontal, temporal and occipital lobes, to the temporal association cortex where this information is fused into a multisensory percept.

Four fMRI studies involved connectivity-based analyses, with two employing Dynamic Causal Modelling (DCM) (Novak et al., 2015; Stickel et al., 2019), one employing psychophysical interaction analysis (Sijben et al., 2018) and one performing graph theoretical network analysis (Ripp et al., 2018). As with olfaction, this reflects a trend towards investigation of network-based interactions underlying multisensory integration. As multisensory integration and crossmodal correspondences require the integration of information from multiple networks, including sensory specific processing networks and memory networks, Sijben et al. (2018) argue that connectivity-based analyses are a better tool to characterise these processes than subtractive analysis models. Similar to Amsellem et al. (2018) and Sijben et al. (2018) included a semi-congruent condition to investigate the impact of semantic congruence on olfactory-visual integration. Their results indicated a differential connectivity of parcellations of the IFG with seed regions from different networks involved in sensory and multisensory processing depending on the degree of congruence between the stimuli. This highlights the crucial role of the IFG in multisensory processing, potentially functioning as a hub for determining the degree of congruence between the stimuli. This supports Ripp et al. (2018) suggestion that IFG supplies odour working memory and semantic information for integration of visual-olfactory information. Increased connectivity with the putamen during congruent and semi-congruent multisensory processing also reflects previous findings of putamen involvement in multisensory integration. Using a connectivity approach, Sijben et al. (2018) were able to go beyond identifying regions involved with visual-olfactory integration, and instead were able to begin to describe the mechanisms of action within these regions.

Rather than using an image to provide visual stimulation, Österbauer et al. (2005) used colours. Odours and colours were presented in unimodal, bimodal congruent or bimodal incongruent form with participants responding as to how well the odour and colour “fit.” Unimodal odour presentation was associated with activity in primary and secondary olfactory regions: bilateral piriformis and amygdalae, putamen, right OFC and left insula. Using colour-odour congruency as an additional parametric modulator, Österbauer et al. identified a network of brain areas exhibiting increasing activity with higher perceived congruence. This network was entirely left lateralised and included OFC, IFG, gyrus rectus and anterior insula. Österbauer et al. also performed an additional contrast to identify regions which express superadditive responses to bimodal congruent stimuli, as described by Calvert et al. (2000, 2001), and Calvert (2001). Regions which express a greater response to bimodal stimulation than the addition of responses to unimodal stimulation conditions (i.e., olfactory-visual > olfaction + visual) are said to express linear superadditivity. Österbauer et al. also included behavioural ratings of colour-odour congruence within their superadditivity model such that BOLD response to colour-odour pairings which were rated as a “very good fit” were modelled as larger than the response to pairings which a “very bad fit.” They identified superadditivity effects of colour-odour stimulation within the SFG, ACC and OFC. Österbauer et al. observation of right OFC involvement in unimodal olfactory processing versus left OFC correlation with colour-odour congruence further supports Broman et al. (2001) theory that the right hemisphere is involved in low-level perceptually-based odour processing, and the left hemisphere associated with higher-level cognitive-based odour recognition and semantic interpretation.

Similarly, Yamashita et al. (2022) investigated colour-odour correspondences. Their use of fNIRS allowed investigation of colour-odour correspondences in a novel immersive paradigm; whilst previous research has typically employed presentation of small-field colour patches or display stimuli, using fNIRS allowed Yamashita et al. to sit participants within a booth illuminated in one of different colours. fNIRS monitoring was performed with two channels covering the left and the right OFC; the study compares the balance of left versus right OFC involvement in each of the stimulation conditions. Perception of pleasant and unpleasant fragrances, and crossmodal colour-odour stimulation were all associated with greater oxyhaemoglobin (HbO) change in the left OFC than right OFC, agreeing with previous findings of greater left hemisphere involvement in higher order cognitive olfactory processes (Broman et al., 2001; Hudry et al., 2014). Crossmodal presentation of odours resulted in greater OFC signal change than crossmodal presentation of odour names. As OFC has been shown to demonstrate superadditivity effects during crossmodal olfactory-visual stimulation (Österbauer et al., 2005), these results indicate that synaesthetically driven crossmodal correspondences are more harmonious than semantically driven correspondences. However, care must be taken when interpreting these results as indications of neural activity. HbO signals are more vulnerable to systemic artefacts which may artificially amplify signal changes and affect interpretation of results (Kirilina et al., 2012; Tachtsidis and Scholkmann, 2016; Dravida et al., 2018). To draw any firm conclusions, the study should be repeated with analysis of both oxy- and deoxyhaemoglobin (HbR) signals to ensure the signal changes are arising from neuronal activity rather than scalp level haemodynamics or other systemic artefacts.

The most common paradigm to investigate crossmodal interactions was a unimodal vs bimodal stimulation task. As expected, unimodal olfactory stimulation was associated with activity within primary and secondary olfactory regions. Bimodal olfactory and visual stimulation was associated with activity in a largely left lateralised network including OFC, IFG, gyrus rectus and insula. These findings further support the theory of lateralised olfactory processing proposed by Broman et al. (2001) that the right hemisphere mediates low level olfactory perceptual processes and the right hemisphere is mediating the higher level cognitive olfactory processes. The involvement of the left IFG reflects previous findings that the left IFG is associated with semantic recognition of odours and hedonic judgements. Superadditive effects were noted in the SFG, ACC and OFC. The involvement of the SFG and ACC provides further support for the role of memory networks within the creation of crossmodal percepts. Additionally, the differential involvement of right OFC in unimodal olfactory and left OFC in bimodal stimulation provides further support for the laterality of olfactory processing proposed by Broman et al. (2001). Functional connectivity analyses highlight the involvement of parietal regions including the IPS and PrC exerting a top-down control over primary sensory regions.

## Discussion

This review analysed current literature to provide an overview of brain regions associated with olfaction, olfactive imagery and crossmodal visual-olfactive correspondences, and the common protocols and methodologies used to research these topics. We now focus our discussion to determine whether fNIRS would be a suitable tool for further research within this field. It is important to note, this review summarises brain regions cited within the reviewed literature for their involvement in olfaction, olfactory imagery and crossmodal visual-olfactory integration, and highlights the potential accessibility of these regions for monitoring via fNIRS technology. Many of the cited regions are large regions with superficial aspects, but also extend deeper within the brain. Where this review hypothesises on the possibility of recording from these regions, this pertains to the superficial aspects of these regions which are within the maximum recording depth of ~1.5 cm from the scalp surface. However, some of the reviewed studies report peak activity associated with the cognitive processes of interest within the deeper aspects of the regions.

This is particularly pertinent in the case of the orbitofrontal cortex (OFC). OFC involvement in olfaction and olfactory imagery interrogated with fMRI usually report peak voxels within the ventral and medial aspects of the OFC; fNIRS technology, however, is only able to record signals from the ventral and lateral aspects of the OFC. Whilst the peak of activity may be beyond the depth of fNIRS monitoring abilities, task-based changes in regional cerebral blood flow may occur across a larger area within the cited region which may be detectable with fNIRS within the superficial aspects of these cortical regions. As summarised in Gunasekara et al. (2022), have used fNIRS to monitor the OFC during olfactory stimulation. Twelve of these studies reported fNIRS signal changes within the interrogated regions of the OFC. However, it must be noted that ten of these studies only report on HbO changes. Due to the susceptibility of this signal to confounding noise, these activations cannot be reliably interpreted as neuronal signal (see Tachtsidis and Scholkmann, 2016 for further information). As such, further investigation would be needed to validate that fNIRS signals recorded in OFC during olfaction are from olfactory-related neurovascular coupling, rather than systemic blood flow changes.

Whilst most primary and secondary olfactory processing regions are subcortical structures, and hence inaccessible with fNIRS technology, a number of superficial tertiary olfactive areas are highlighted within the literature as being involved in olfactory perception and higher-level olfactory processes. Whilst these functional centres are not unique to olfactive processes, many report reliable activation within olfactory tasks. Olfactory perception tasks reliably activated the piriform cortex, as well as the insula and cingulate gyrus (see Table 2); all commonly cited primary and secondary sub-cortical olfactory regions. Additionally, all olfactory studies found activation in at least one cortical region accessible to fNIRS. The most commonly identified region was the OFC, a secondary olfactory processing region, cited in ten papers. Other commonly cited regions included IFG, PrC, SMA, PrCG, and DLPFC. Using a well-established task which is known to involve olfactory processing, olfaction can be studied using fNIRS, with regions of interest accessible in the frontal and parietal lobes. As summarised in Gunasekara et al. (2022), multiple studies have already used fNIRS to study olfaction. These studies predominantly considered prefrontal regions of interest.

As fNIRS devices are portable and wearable and do not require a specialist shielded room or strong magnetic environment, as with EEG and fMRI, fNIRS technology lends itself to multi-modal monitoring. As demonstrated by Hucke et al. (2023), fNIRS can be combined with EEG allowing insights into olfactory processing beyond what would be possible by a single monitoring modality alone. fNIRS can also be combined with physiological measurements such as cardiac and blood pressure monitoring, breathing monitoring, electrodermal monitoring and plethysmography monitoring. As hedonic odour processing, and the highly emotive nature of olfactive memory are common themes within olfactory research, application of fNIRS monitoring with accompanying physiological measurements may be able to provide new insights within this field. Royet et al. (2003) investigated emotional responses to pleasant and unpleasant odours using fMRI with accompanying electrodermal, plethysmography and breathing monitoring to detect covert emotional responses. Whilst multimodal monitoring in this way is possible with existing neuroimaging techniques, this often requires specialised and expensive systems due to the restrictive environments required for these imaging methods. The ease of including multimodal physiological measurements alongside fNIRS may allow future studies to similarly study the impact of covert emotional responses on olfactory hedonic judgements, and olfactory memory encoding and recall. In a similar vein, applying fNIRS with accompanying multimodal physiological monitoring to the study of olfactory imagery and crossmodal visual-olfactory interactions may allow for novel insights into the role of emotional association in the recall and imagination of odours, such as imagining personally nostalgic odours, and the multisensory integration of emotionally charged odours with congruent visual cues. Accompanying electrodermal and plethysmography recordings may vary between fMRI and fNIRS due to the different postures during monitoring, signal change findings should remain consistent between the two modalities.

Furthermore, applying fNIRS technology to a paradigm such as described in Leclerc et al. (2019) may result in significant findings between sham and tDCS conditions which were not found in their present study. Leclerc et al. applied real or sham tDCS to the SMA prior to performing an imagery and source memory task. They hypothesised tDCS would result in neuromodulatory effects to the SMA which would alter source memory and imagery generation. However, they found no significant effects of tDCS on imagery or source memory performance and concluded that the neuromodulatory effects may have been lost to washout before scanning could be performed. As fNIRS technology can be used in conjunction with tDCS (Muthalib et al., 2013; McKendrick et al., 2015), repeating Leclerc et al. paradigm with fNIRS rather than fMRI could allow neuromodulatory effects of tDCS to be investigated without losing them to washout during preparation and set-up of the scanner.

Olfactory imagery has been consistently demonstrated to recruit olfactory regions including the piriform cortex, insula, hippocampus, amygdala and OFC (Djordjevic et al., 2005; Bensafi et al., 2007; Plailly et al., 2011; Royet et al., 2013a,b; Flohr et al., 2014; Schienle et al., 2017). All reviewed studies reported activation in at least one of these regions during olfactory imagery. Multiple reviewed studies also cited additional superficial cortical regions activated during olfactory imagery. Leclerc et al. (2019) identified a mostly left-lateralised network of cortical regions exhibiting greater activation during olfactory imagery than during olfactory perception. Regions included left DLPFC, IFG, IPS, angular gyrus and pre-SMA, and right frontal pole and IFG. Each of these regions were cited in at least one other reviewed article. The left DLPFC, IFG, IPS, angular gyrus and pre-SMA are regions which have also been implicated in modality-general imagery (McNorgan, 2012). McNorgan (2012) analysis identified four left lateralised regions recruited exclusively by olfactive imagery. Of these four regions, only the cluster in the left SPL would be accessible using fNIRS. Left DLPFC, IFG, IPS, angular gyrus and pre-SMA can be monitored using fNIRS to identify rCBF changes within these regions during olfactive imagery, but care must be taken to ensure the task is evoking olfactory mental imagery, and not involuntary imagery across other, more dominant, sensory modalities such as visual imagery. Using a task such as Han et al. (2022) which used visual imagery generation as a control condition in an olfactory imagery task could allow for the subtraction of activations associated with involuntary visual imagery generation from olfactory imagery activation. Alternatively, asking participants to self-report whether they experienced co-occurring imagery across other modalities when generating an olfactive image could be used to ensure the task is evoking olfactory imagery.

fNIRS can also be used to investigate lateralisation of function across hemispheres, a prominent topic of investigation across all three of the reviewed research domains. The degree of lateralisation of activity can be evaluated by calculating the laterality index, similar to methods used by Zhou et al. (2019), where laterality is equal to left hemispheric activity minus right hemispheric activity, divided by combined left and right hemisphere activity (Ishikawa et al., 2014). This results in a laterality index score between [−1 to 1] where negative values represent greater right hemispheric lateralisation and positive values represent greater left hemispheric lateralisation. In this manner, laterality can be assessed on a whole hemisphere basis, on particular regions, or on a single channel-wise basis. Applying fNIRS to any of these research domains, laterality can easily be studied in this way. For example, Leclerc et al. (2019) identified that the olfactory imagery network is largely left lateralised. Repeating Leclerc et al. paradigm and using a lateralisation analysis should result in a positive laterality index between the left and right hemispheres on a whole brain level, and positive laterality indices between channels covering the left and right DLPFC, IPS, angular gyri and pre-SMA. Additionally, comparing the left and right IFG should result in a positive laterality index, but to a lesser degree, and comparing the left and right frontal poles should result in a negative laterality index during olfactory imagery.

fNIRS technology applied to a unimodal vs. bimodal paradigm could also be used to evaluate linear superadditivity during bimodal olfactory-colour stimulation as described in Österbauer et al. (2005). As with fMRI, amplitude of signal change can be evaluated with fNIRS to identify regions which express greater activation to bimodal olfactory-colour stimulation than the sum of unimodal olfactory and unimodal colour stimulation. Using a paradigm such as Österbauer et al., unimodal odour stimulation should result in detectable signal changes in the right OFC. Bimodal presentation of odour and colours should result in detectable signal changes in left IFG, frontal operculum and temporal pole. Additionally, these regions should exhibit increasing activity with higher perceived congruence. Finally, superadditive signal increases should be detectable in the left SFG. However, as seen in Yamashita et al. (2022), application of fNIRS technology could allow for extension of Österbauer et al. paradigm beyond colour patches to create a more immersive paradigm by placing participants in a coloured booth, or allowing participants to freely move between environments with different odour and colour combinations; this can allow for investigation of superadditive effects of olfactory-colour stimulation within more ecologically valid environments.

As fNIRS signals are extremely susceptible to contamination by physiological noise, block designs are commonly employed to maximise statistical power (Tie et al., 2009; see also Friston et al., 1999; Brockway, 2000). Whilst event-related designs can be used with fNIRS, they have less statistical power than blocked designs, and as such, require a greater number of participants and repetitions to increase this power (Tie et al., 2009). Presentation of olfactory stimuli in a rapid, time-locked procession as is required for event related designs required highly specialised equipment. Furthermore, for use with fMRI and EEG, this equipment must be specifically designed to meet the environmental requirements of these modalities. Use of block designs in fNIRS removes the need for rapid event-related stimulus presentation. However, care must still be taken in the consideration of odour delivery methods to ensure odour presentation can still be time-locked, odours can persist at an even intensity across block length, and that odours do not persist beyond the block length. As such, a specialised odour delivery tool may still be required. Alternatively, the portability of fNRIS could allow for the creation of novel paradigms which could present different odours to the participant by the use of differently fragranced rooms, for example. With the advancement of tools for statistical analysis of fNIRS signals collected from naturalistic paradigms, odours could be delivered in an even more ecologically valid method such as creating “odourscapes” in which the participant could move freely.

The portability of fNIRS devices could also allow future novel paradigms to be developed which allow participants to explore olfaction, odour imagery and crossmodal interactions whilst moving freely in an immersive environment. Indeed, Yamashita et al. (2022) paradigm reflects a move in this direction by applying fNIRS technology to investigate crossmodal colour-odour correspondences in an immersive lighting environment. Future research could investigate the perception or imagination of odours, or crossmodal visual-odour correspondences within naturalistic environments with rich ecological validity. Neuroimaging study design usually requires stringent time-locked events. However, advanced analytic approaches, such as Automatic IDentification of functional Events (AIDE) method, can allow for a brain-first approach to identify event onsets from real-world fNIRS neuroimaging data (Pinti et al., 2017). This can allow for flexible self-paced paradigms without the need for stringent time constraints, further increasing the ecological validity of the study.

Whilst there are a number of neuroimaging and behavioural paradigms which can be adapted for research using fNIRS, and scope for the development of novel naturalistic paradigms, care but be taken when designing these studies to ensure the reliability, validity and reproducibility of any findings. As fNIRS signals are recorded at the scalp level, they are vulnerable to contamination from systemic noise (see Tachtsidis and Scholkmann, 2016 for full review). Physiological noise sources such as heart rate, breathing, mayer waves and scalp haemodynamic changes can be characterised using short-separation channels and physiological monitoring, and these components can be regressed from the fNIRS signal. Using additional physiological monitoring of respiration characteristics is particularly pertinent in olfactory and odour imagery research as both olfaction and odour imagery are associated with modulations to breathing (Bensafi et al., 2005; Mainland and Sobel, 2005; Kleemann et al., 2008; Rinck et al., 2008). Additionally, study designs should avoid stimulation frequencies which overlap with systemic oscillations such as the respiration rate (~0.3 Hz) and the mayer wave (~0.1 Hz) as these can artificially amplify the fNIRS signal. As described above, jittering rest periods can also help to avoid synchronisation with systemic fluctuations. It is also crucial to investigate both oxy- and deoxyhaemoglobin signals. During a haemodynamic response to support neuronal activity, the concentrations of HbO increases and HbR decreases due to the oversupply of blood flow to support neuronal function. As such, the fNIRS signals for HbO and HbR concentration should be anticorrelated within the active region. Failure to investigate both parameters could lead to misinterpretation of signal changes from systemic sources as evidence of neuronal activity (for further information regarding the best practices for fNIRS research and publications, see Yücel et al., 2021).

## Conclusion

Olfaction, olfactive imagery and crossmodal visual-olfactory integration are all associated with activation in widespread cortical regions across frontal, parietal, temporal and occipital lobes. Many of the regions functionally activated during these processes would be accessible for monitoring using fNRIS. Additionally, many of the common paradigms and protocols would be suitable for conducting research with fNRIS technology. Furthermore, fNIRS suitability for use in naturalistic settings may allow for development of new research paradigms in naturalistic settings with greater ecological validity than previously available neuroimaging techniques.

## Author contributions

EB: Writing – original draft, Writing – review & editing. AL: Writing – review & editing. GG: Writing – review & editing. NG: Writing – review & editing. EP: Writing – review & editing. EK: Writing – review & editing. MJ: Writing – review & editing. IT: Writing – review & editing.
